# Resource: A compendium of HLA types and expression in pediatric cancer models

**DOI:** 10.1016/j.isci.2025.113887

**Published:** 2025-10-28

**Authors:** Yiwen Guan, Ishika Mahajan, Vikesh Ajith, Dingyin Sun, Isaac Woodhouse, Tima Shamekhi, Pouya Faridi, Ron Firestein, Claire Xin Sun

**Affiliations:** 1 Centre for Cancer Research, Hudson Institute of Medical Research, Clayton, VIC 3168, Australia; 2 Department of Medicine, School of Clinical Sciences, Monash University, Clayton, VIC 3168, Australia; 3 Monash Proteomics and Metabolomics Platform, School of Clinical Sciences, Monash University, Clayton, VIC 3168, Australia

**Keywords:** molecular biology, immunology, cancer

## Abstract

Cancer immunotherapy has revolutionized treatment by leveraging the immune system to recognize and destroy tumor cells, offering a promising, less toxic option for pediatric patients. A key component of this response is antigen presentation, which depends on accurate human leukocyte antigen (HLA) typing and expression. However, immune-focused resources for pediatric cancers remain limited. In this study, we present a comprehensive immunogenomic resource covering 231 cancer cell lines and 56 tumor-associated fibroblast cell lines from the Childhood Cancer Model Atlas (CCMA). We inferred high-resolution HLA types, predicted neoantigens arising from somatic single nucleotide variants, gene fusions, and splicing isoforms across multiple tumor types, and quantified HLA expression levels. We also explored immune escape mechanisms, including loss of heterozygosity and allele-specific expression loss of HLA genes. This publicly accessible dataset provides critical insight into the immune landscape of pediatric cancers and serves as a foundational tool for immunotherapy development.

## Highlights

• HLA types profiled across 200+ pediatric cancer cell line models
• Neoantigen candidates predicted by mutations, fusions, and splicing events
• Transcriptional landscape of antigen processing and presentation pathways
• Resource enables immunogenomic discovery in pediatric cancer research

## Graphical abstract

**Figure:**
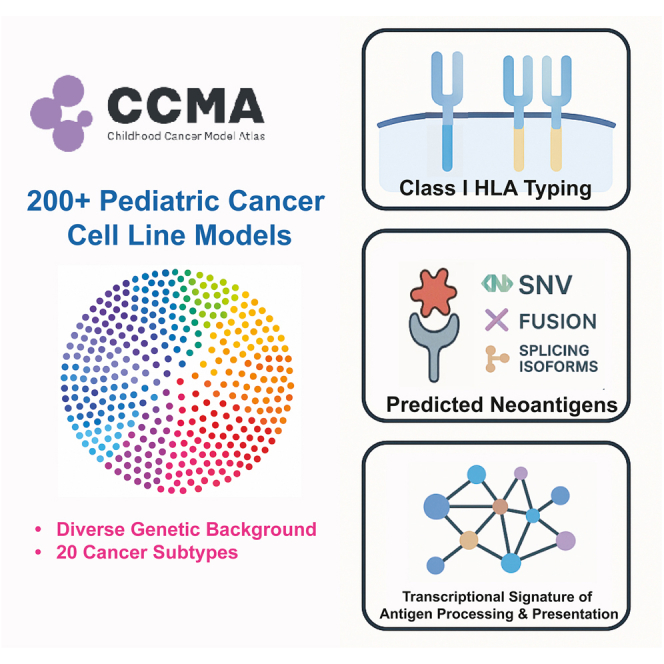

## Introduction

Cancer immunotherapy is a transformative approach that enhances the body’s immune system to recognize and eliminate tumor cells. It is emerging as a promising strategy in pediatric cancers.^1^^,^^2^^,^^3^ Recent advancements, including T cell receptor (TCR) based cell therapies and personalized cancer vaccines incorporating precision neoantigens, have significantly improved patient outcomes across various cancer types.^4^^,^^5^^,^^6^^,^^7^ Particularly, advances in cancer vaccines have elicited *de novo* T cell responses against tumor antigens, offering further promise in combating diverse malignancies.^8^ TCR therapy targets tumor cells by using T cells engineered to express antigen-specific TCRs, which recognize peptide and major histocompatibility complex (MHC) complexes presented on the tumor cell surface. This approach enables the targeting of intracellular tumor-derived peptides displayed via MHC molecules, thereby broadening the antigen spectrum accessible to immunotherapy.^5^^,^^9^^,^^10^^,^^11^ Notably, these strategies are also being translated into pediatric oncology, where TCR-based therapies and neoantigen-targeted vaccines are beginning to show encouraging clinical responses in select pediatric solid tumors and hematologic malignancies.^12^^,^^13^

Tumor antigen presentation plays a central role in immune recognition, acting as a bridge between tumor cells and the immune system.^14^^,^^15^ The canonical antigen presentation pathway begins with proteins being degraded into peptides by the proteasome, followed by the loading of these peptides onto MHC I molecules. In humans, the MHC is also known as the human leukocyte antigen (HLA) system. HLA molecules, encoded by HLA genes on chromosome 6, are essential in the presentation of antigens to T cells, thus influencing immune recognition and tumor evasion. HLA typing is a fundamental aspect of immunogenetics, particularly in its restriction of neoantigen presentation. Neoantigens include viral, mutated, and nonmutated antigens newly and preferentially presented on tumor cells.^16^ The presentation of neoantigens on the HLA complex is the key mediator of the antitumoral T cell responses. Disruption of HLA molecules (somatic HLA loss of heterozygosity (LOH) or allele-specific expression loss (ASE loss)) has also been shown to limit the efficacy of neoantigen presentation and therefore, influences cancer response to checkpoint blockade immunotherapy.^17^^,^^18^^,^^19^^,^^20^^,^^21^ Accurate identification of neoantigens and their presenting HLA alleles is a key bottleneck in advancing neoantigen-restricted immunotherapies.

The Childhood Cancer Model Atlas (CCMA) initiative is at the forefront of pediatric cancer research, providing extensive multi-omics profiling of the largest collection of pediatric cancer cell line models (http://vicpcc.org.au/dashboard).^22^ While emerging studies have started to translate advances in adult cancer immunotherapies to pediatric settings, there is growing promise due to the distinct and diverse neoantigen landscape observed in childhood cancers.^23^ Cell lines serve as essential preclinical models and powerful tools in the pre-clinical development of innovative immunotherapy treatments. Accurate HLA typing is a critical part, enabling researchers to choose the appropriate model to investigate the interaction between cancer cells and the immune system. The integration of comprehensive HLA typing, predicted neoepitope, and assessments of immune presentation expression activity provides valuable insights into the immunological characteristics of these cell lines, thereby advancing personalized medicine strategies.

## Results

### The characterization of human leukocyte antigen genotypes

The CCMA hosts the largest single-site collection of pediatric cancer cell line models. The 215 pediatric cell lines reported in this study span nine cancer types, including pediatric high-grade gliomas (pHGG, *n* = 114), which consist of 67 diffuse midline glioma with H3K27M (DMG-H3K27M), 36 high-grade glioma with wildtype H3 (HGG-H3WT), 10 diffuse hemispheric glioma with H3G34 R/V mutation (DHG-H3G34), and 1 astrocytoma. Bone and soft tissue sarcomas (*n* = 34) include 12 Ewing sarcoma, 11 osteosarcoma, 8 rhabdomyosarcoma, 2 chordoma, 1 inflammatory myofibroblastic tumor (IMFT). Embryonal tumors (*n* = 38) include 20 atypical teratoid/rhabdoid tumors (ATRT), 11 medulloblastoma, 4 embryonal tumors with multilayered rosettes (ETMR), 2 malignant rhabdoid tumors (MRTs), and 1 pineoblastoma. Ependymal tumors (*n* = 9) include 9 ependymomas, and neuroendocrine tumors (*n* = 17) include 17 neuroblastomas. Other rare cancer types (*n* = 3) consist of 1 central nervous system sarcoma, 1 choroid plexus carcinoma, and 1 nerve sheath tumor. Additionally, there are 56 tumor-associated non-malignant cell lines, and 16 adult high-grade gliomas (aHGG) included in the cohort. Among the 287 unique cell lines, 174 have been previously studied, while 113 are newly established models sourced from local hospitals and collaborators. New RNA-seq data (*n* = 103) and WGS data (*n* = 93) are presented in this study along with previously published CCMA datasets.^22^

**Figure 1:**
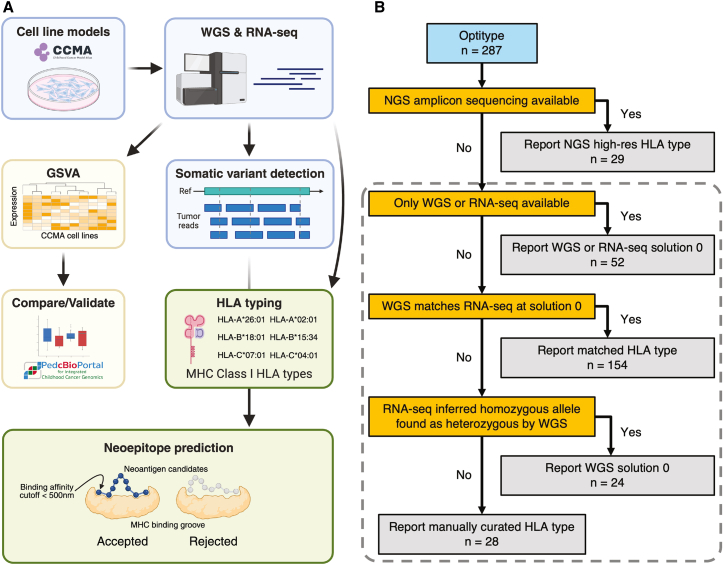
Computational workflow and data integration for HLA expression, typing, and neoepitope prediction in the CCMA (A) WGS and RNA-Seq data from 287 cell lines were processed for GSVA and Class I HLA typing. HLA pathway activity scores were compared to primary tumors (OpenPedCan). The cell line-specific somatic mutations and HLA types were processed to predict potentially effective neoepitopes (Figure created with BioRender.com). (B) Decision-making process for selecting the optimal HLA types. The final HLA types were determined hierarchically by each criterion based on data availability. HLA types inferred from Optitype were denoted by the dash-line box. See also Table S1.

**Figure 2:**
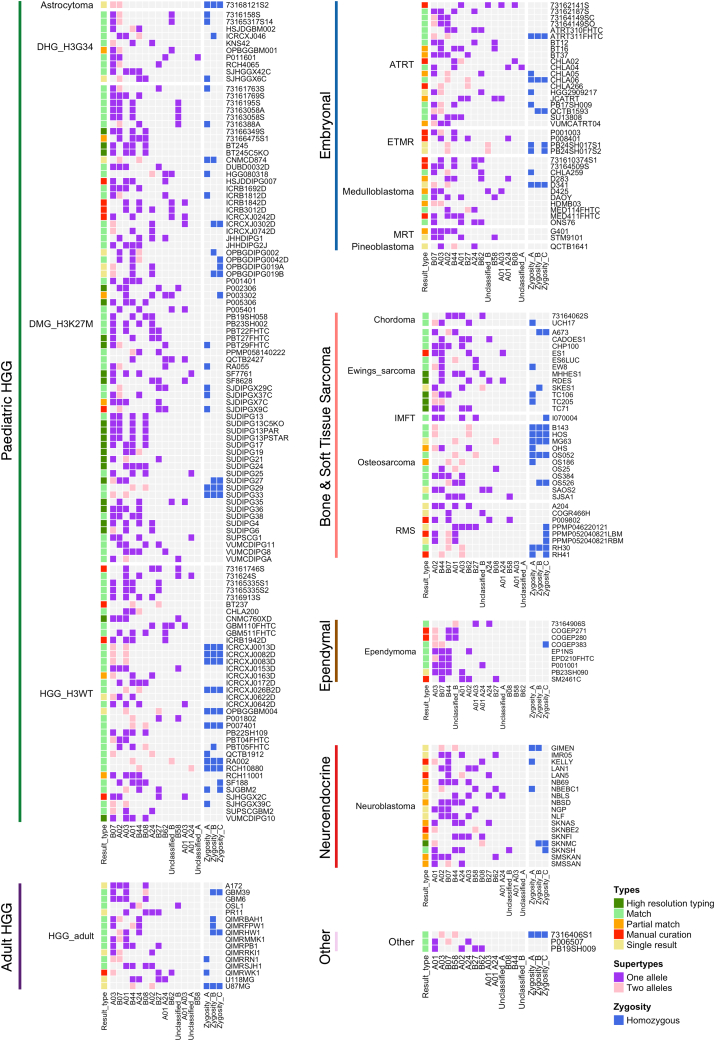
Tile plot for the CCMA HLA types Tile plot shows data integration, supertypes, and zygosity for the CCMA models. Subplots were grouped by cancer type and clustered by CCMA cancer class. Data, including result type (high-resolution typing, match, partial match, manual curation, and single result), supertypes (“One” representing allele and “Two” representing alleles), and zygosity (homozygous) for each model, were color-coded as indicated. See also Figure S1 and Table S2.

**Figure 3:**
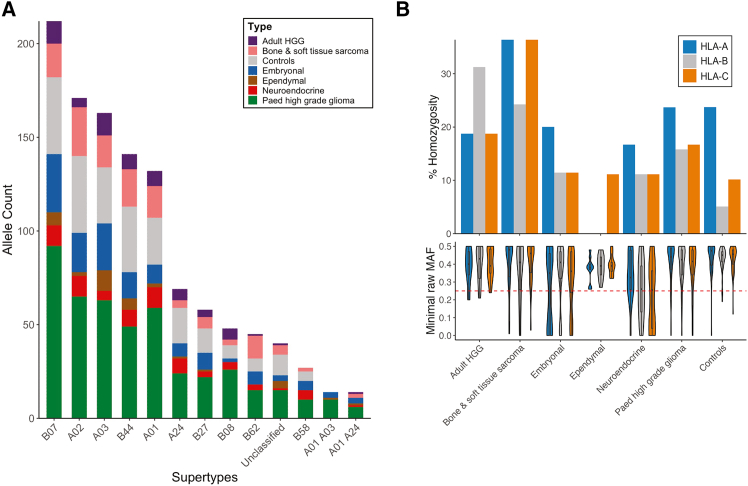
Distribution of HLA supertypes, zygosity, and ASE loss events across the CCMA (A) Stacked bar chart shows the HLA allele count of CCMA models across 12 supertypes or unclassified. HLA supertypes were defined according to Sidney et al., 2008.^27^ (B) Comparison of HLA class I homozygosity percentage and ASE across three genes in cancer types. HLA class I ASE is represented by the minimal raw minor allele frequency (MAF) among the three HLA class I genes. A reference line MAF = 0.25 was labeled in red. See also Table S2.

**Figure 4:**
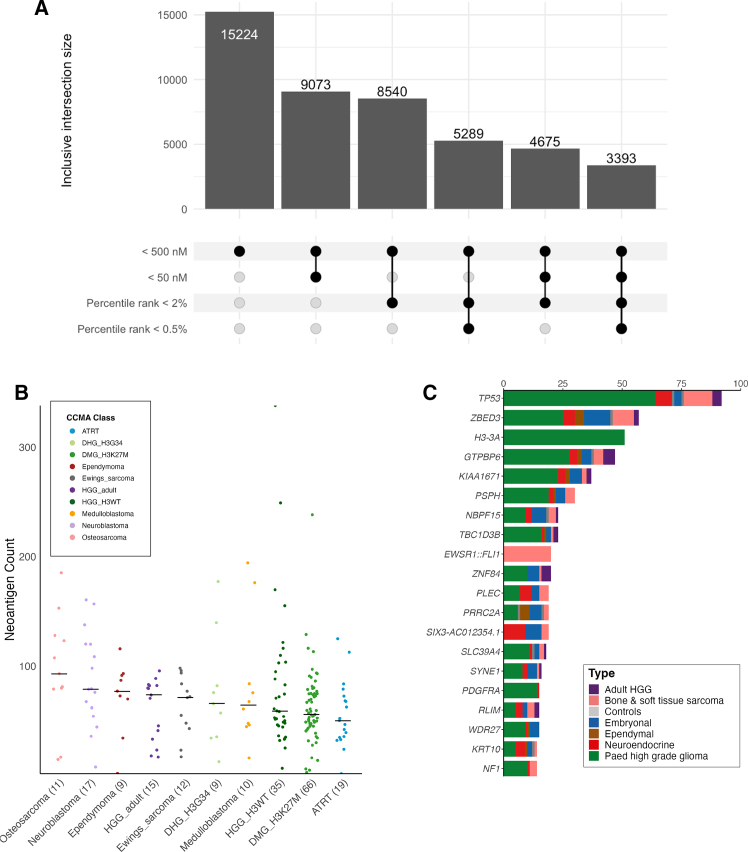
Predicted neoepitope profiles for CCMA (A) Subgroup intersections between filtering criteria for predicted neoepitopes. Thresholds were determined by three types of prediction algorithms, favoring different epitope qualities (binding and elution). For binding and elution criteria, weak binders (IC50 < 500 nM; percentile ranking <2%), and strong binders (IC50 < 50 nM; percentile ranking <0.5%) were considered. (B) Distribution of predicted neoepitopes with IC50 < 500 nM across 11 CCMA cancer classes. The median number of neoepitopes for each class was labeled. (C) Top 20 recurrently mutated genes within the predicted neoepitope catalog. Distribution of the genes across different cancer types was shown as labeled. See also Figure S2 and Table S3.

Using high-resolution HLA typing and WGS/RNA-seq HLA typing with Optitype,^24^ we determined 4-digit class I HLA types and characterized the level of HLA expression and antigen presentation pathways for 287 CCMA cell lines (Figure 1A; Table S1.1). Additionally, neoepitopes were predicted for 231 cancer models using pVACtools^25^ based on the somatic variants identified by WGS and RNA-seq (Figure 1A; Table S1. HLA class I typing for CCMA cell line models, related to Figure 1, Table S2. Accuracy of HLA typing and zygosity, related to Figures 2 and 3, Table S3. Predicted neoepitopes for CCMA cell line models, related to Figure 4). The reported HLA types were determined using a robust method illustrated in Figure 1B. A total of 29 cell lines were assessed using the NGS high-resolution HLA typing assay, deemed the gold standard. Of the remaining 258 lines, 206 had both WGS and RNA-seq data available for typing, 17 had only WGS-based inference, and 35 had only RNA-seq based inference. Among the 52 lines with single source typing results, the optimal solution was reported (Solution 0 from the Optitype algorithm). Of the remaining 206 models with dual-source results, 154 showed complete concordance between the optimal WGS and RNA-seq typing solutions (Solution 0). In 24 models, homozygous HLA types were identified by RNA-seq, while the corresponding WGS types were heterozygous (more details in the next section). In these cases, the WGS Solution 0 results were reported. The remaining 28 models exhibited non-concurrent HLA typing, and their HLA types were subjected to manual curation. Among these, 13 models achieved matching HLA types at 2-digit resolution (Table S1.2). The mean concordance rate of all the HLA alleles between the 28 mismatched cell lines was 82.1% at 2-digit resolutions and 69.1% at 4-digit resolutions (Figure S1A).

### The human leukocyte antigen supertypes and zygosity for the Childhood Cancer Model Atlas models

HLA supertypes represent distinct groups of HLA alleles that share peptide-binding specificities.^26^^,^^27^ These supertypes have been widely used to guide neoepitope prediction across diverse HLA alleles,^25^ and to explore immunotherapy outcomes.^28^^,^^29^ The CCMA cohort exhibited a strong representation of HLA supertypes, which is important in immunotherapy studies due to their broad population coverage (Figure 2). Supertypes for HLA genes A and B were categorized for all cell lines. The most prevalent supertypes in the examined cell lines were A02, A03, and A01 for gene *HLA-A* (173, 166, and 132 of 572 alleles, respectively) and B07 and B44 for gene *HLA-B* (214 and 141 of 574 alleles, respectively) (Figures 2 and 3A).

Heterozygous HLA sequences provide an advantage by enabling the presentation of diverse immunopeptides.^30^^,^^31^ In contrast, homozygous HLA genotypes are associated with poorer clinical outcomes in cancer immune checkpoint blockade (ICB) therapy.^32^^,^^33^ Recent studies revealed that somatic HLA LOH is a common cancer immune evasion strategy.^17^^,^^34^ LOH at the HLA locus typically arises from irreversible genomic alterations on chromosome 6. In parallel, ASE loss can occur at the transcriptomic level, reflecting selective silencing or downregulation of one HLA allele. Both somatic HLA LOH and ASE loss have been associated with decreased survival, immune evasion, and poor therapeutic responses.^18^^,^^19^^,^^35^ In the CCMA cohort, we observed a significant proportion of homozygous HLA types (Figure 2), which we suspect may be attributed to HLA LOH. We then examined the homozygosity frequency of HLA class I genes across different cancer types in CCMA (Figure 3B; Table S1). The homozygosity frequencies for *HLA-A* ranged from 0% in ependymal to 36% (12 out of 33) in bone and soft tissue sarcoma models (Figure 3B). For *HLA-B*, frequencies ranged from 0% in ependymoma to 31% (5 out of 16) in aHGG models, while the homozygosity frequencies of *HLA-C* ranged from 11% (2 out of 19) in neuroendocrine to 36% (12 out of 36) in bone and soft tissue sarcoma models (Figure 3B).

Furthermore, we identified discrepancies between HLA types inferred from WGS and RNA-seq in 24 models. Specifically, these models exhibited heterozygous HLA types in WGS but appeared homozygous in RNA-seq, with only one of the alleles from the heterozygous pair being expressed. This pattern suggests that the expression of the other allele was suppressed at the transcriptomic level, suggesting potential ASE loss events. To investigate, we used *arcasHLA-quant* to quantify HLA class I ASE for all cell line models with available RNA-seq data. A total of 79 samples were identified with ASE loss events, defined as minor allele frequency (MAF) < 0.25^17^^,^^19^ observed in at least one of the three HLA class I genes. Specifically, ASE loss was detected in 38 samples for *HLA-A*, 37 samples for *HLA-B*, and 36 samples for *HLA-C*. No significant differences in ASE loss were observed among the three genes within each cancer type (Figure 3B). Notably, neuroendocrine cancers exhibited the lowest median MAF across all three genes (median MAF = 0.31). We found that combined pediatric cancer types exhibited more ASE loss events than control samples (adjusted *p*-value <1.7 × 10).

To test the robustness of HLA typing with WGS and RNA-seq, we compared Optitype results to NGS high-resolution typing. From 29 lines with high-resolution typing results available, there were two sets of isogenic cell line families (BT245 and BT245C5KO; SUDIPG13, SUDIPG13C5KO, and SUDIPG13PSTAR). Only parental models were considered for accuracy calculations (BT245 and SUDIPG13). Of the 26 unique models, 25 achieved at least 2-digit accuracy, with mean accuracies of 96.8% and 94.2% at 2-digit and 4-digit resolutions, respectively (Table S2.1; Figure S1B). Only one model, VUMCDIPG10 (three true heterozygous loci), was incorrectly predicted by Optitype as homozygous by WGS at the *HLA-A*, *HLA-B*, and *HLA-C* loci. Therefore, this line was reported as undetermined. The overall zygosity prediction accuracy was 96.2% (Table S2.2; Figure S1C). Most cell lines had complete matches, while four had minor mismatches at four-digit resolution. Overall, WGS and RNA-seq based HLA inference achieved high accuracy comparable to the standard high-resolution NGS typing method.

### The predicted neoepitope profiles in the Childhood Cancer Model Atlas

Mutated neoantigens are tumor-specific antigens due to somatic mutations. Since neoantigens include shared neoantigens (antigens expressed *de novo* or overexpressed) and antigens from integrated viral open reading frames.^16^ In this study, we use the term “neoepitope” to specifically denote the mutated peptide sequences, resulting from somatic missense mutations, in-frame indels, frameshift mutations, gene fusions, or dysregulated RNA splicing that are presented by HLA molecules. Effective neoantigens presented by the MHC can activate CD8^+^ and CD4^+^ T cells, triggering tumor-specific immune responses.^15^ Neoantigens serve as key targets in cancer immunotherapies and personalized treatments. While extensive neoantigen research has been conducted in adult cancers such as lung cancer,^36^ pancreatic cancer,^37^ and melanoma,^38^ there remains a significant knowledge gap in pediatric cancers. Using the pVACtools epitope pipeline with selected prediction algorithms and prioritization filters, we identified neoepitope candidates for 231 cell line models in the CCMA based on their WGS and RNA-seq profiles. We employed a robust pipeline consisting of three binding prediction models (NetMHCpanBA, MHCflurry, and MHCnuggetsI) and two elution prediction models (NetMHCpanEL and MHCflurryEL) to systematically prioritize neoepitopes. For initial exploratory analysis, we applied an IC50 binding threshold of <500 nM to filter candidates.

Across the 231 models spanning ten cancer types, we identified a total of 15,224 putative neoepitope candidates with an IC50 score below 500 nM (Figure 4A), comprising 11,462 from single nucleotide variants (SNVs), 652 from splicing variants, and 3,110 from fusion variants. While 9,073 (59.6%) were considered strong binders with an IC50 score below 50 nM (Figure 4A; Table S3.1). The distribution of neoepitopes per model was well-balanced across cancer types (Figure 4B). To further incorporate the biological relevance of peptide processing, TAP-mediated peptide transport, and MHC presentation, we applied percentile filters (<2% and <0.5%) from elution prediction models to all neoepitopes meeting the IC50 < 500 nM threshold. Out of the 15,224 candidates, 8,540 (56.1%) and 5,289 (34.7%) neoepitopes had a percentile rank of <2% and <0.5%, respectively. We observed 3,393 (22.3%) neoepitope candidates exhibiting high predicted likelihood of elution in addition to strong binding affinity (Figure 4A). We further analyzed the distribution of these 3,393 filtered neoepitopes across HLA allele types (Figure S2). The most frequently predicted bound HLA allele was HLA-A∗02:01, accounting for 362 neoepitopes, followed by HLA-B∗07:02 (*n* = 230) and HLA-C∗07:02 (*n* = 198). The number of predicted neoantigens presented by common HLA allotypes is a reflection of their prevalence in the general population,^39^ indicating the applicability of the CCMA cell line collection.

The top 20 recurrent genes from the 15,224 predicted neoepitopes are shown in Figure 4C. Neoepitopes were frequently contributed by SNVs of *TP53* and *H3-3A*, *PSPH* for splicing variant, and *EWSR1*::*FLI1* fusion variations. As expected, *H3-3A* predicted neoepitopes (H3.3K27M) were exclusively observed in the pHGG cancer type. The H3.3K27M mutation was the major contributor to predicted neoepitopes, followed by H3.3G34R and G34V. *TP53* predicted neoepitopes were observed across all cancer types except ependymal models. No dominant contributing variation was observed, as diverse mutations resulted in distinct and unique predicted neoepitopes. For splicing variants, the *PSPH*: c.275+1del mutation was considerably more common than other splice variants. For fusions, the *EWSR1*::*FLI1* fusions were frequently and only found in bone and soft tissue sarcomas, specifically Ewing sarcomas, as their pathogenic hallmark. Other known recurrent cancer genes with ≥10 occurrences include *PLEC*, *PDGFRA*, *NF1, ARID1A, BCOR,* and *ACVR1* (Table S3). These mutated genes predicted to generate effective neoepitopes were recurrently observed across diverse cancer types, suggesting their potential as therapeutic targets.

To support our neoepitope prediction approach, we cross-referenced predicted neoepitopes from the CCMA with those previously reported in the Pediatric Cancer Genome Project cohort by Chang et al., 2017.^23^ In this study, they analyzed 540 pediatric tumor samples across 23 cancer types with paired normal samples, which include 128 solid tumor samples and 123 CNS tumor samples. From a subset of 2453 neoantigens identified in the solid and CNS tumor sub-cohort, we commonly predicted four neoepitopes (Table S3.4) from eight cell line models, of which two neoepitopes are associated with recurrent mutations *H3-3A* K27M and *ALK* F1174L. We further compared the predicted neoepitope profiles in our dataset with validated neoepitopes from the Immune Epitope Database (IEDB).^40^ Ten peptides with various HLA restrictions and T cell assays were found in the predicted neoepitopes of 13 cell line models; all ten validated epitopes passed at least one of the binding or elution model filters (Tables 1 and S3.5). Validated targets included widely recognized cancer antigens (e.g., TP53, KRAS, NRAS, EGFR). Among these, the hallmark mutation of DMG, *H3-3A* K27M, and its derived neoepitope H3.3K27M_26-35_ (RMSAPSTGGV), were predicted in seven models. This neoepitope has been the subject of significant yet controversial immunogenicity research, underscoring the clinical relevance of these models for the validation of presence using an immuno-peptidomics approach and functional studies.^41^^,^^42^^,^^43^^,^^44^^,^^45^^,^^46^^,^^47^ In addition, six neoepitopes from different *TP53* mutations were predicted in nine cell line models. Several neoepitope and HLA allele combinations from CCMA neoepitope predictions were validated with MHC ligand assays. Overall, the CCMA neoepitope catalog provides an ideal cohort of preclinical models, serving as a foundation for hypothesis-driven studies of neoantigen-targeted T cell therapies in pediatric cancers.

**Table 1:** Peptides from IEDB labeled “neoepitopes” predicted in the CCMA cohort

Gene	Mutation	Predicted MT sequence	WT sequence	HLA restriction	IC50 affinity (nM)	Epitope length	Assay type (Positive)	CCMA stripped ID with predicted HLA allele
EGFR	Ala289Val	YSFG**V**TCV	YSFGATCV	HLA-C∗02:02	8.5	8	T cell Assay	ICRCXJ0642D (HLA-C∗02:02)
KRAS	Gly12Asp	GA**D**GVGKSAL	GAGGVGKSAL	HLA-C∗05:01	39.1	10	MHC Ligand Assay (HLA-C∗08:02; **HLA-C∗05:01**; HLA-B∗07:02; HLA-C∗03:04) & T cell Assay	JHHDIPG2J (HLA-C∗05:01)
KRAS	Gly12Asp	VVVGADG**V**GK	VVVGAGGVGK	HLA-C∗03:03	77.5	10	MHC Ligand Assay (HLA-A∗11:01; HLA-A∗03:01; HLA-A∗68:01) & T cell Assay	NBEBC1 (HLA-C∗03:03)
NRAS	Gln61Lys	ILDTAG**K**EEY	ILDTAGQEEY	HLA-A∗01:01	39.7	10	MHC Ligand Assay (**HLA-A∗01:01**; HLA-A∗01:191; HLA-B∗15:01; HLA-C∗08:02) & T cell Assay	73166475S1 (HLA-A∗01:01)
TP53	Arg248Trp	MN**W**RPILTI^a^	MNRRPILTI	HLA-C∗06:02	57.3	9	MHC Ligand Assay (HLA-A∗24:02)	7316388A (HLA-C∗06:02) P001401 (HLA-C∗03:03)
				HLA-C∗03:03	7.0			
TP53	Arg273Cys	EV**C**VCACPGR^a^	EVRVCACPGR	HLA-A∗68:01	22.9	10	MHC Ligand Assay (HLA-A∗33:03)	SUDIPG27 (HLA-A∗68:01) SJGBM2 (HLA-A∗68:01) P003302 (HLA-A∗31:01)
				HLA-A∗31:01	496.3			
TP53	Arg248Gln	SSCMGGMN**Q**R^a^	SSCMGGMNRR	HLA-A∗11:01	171.2	10	MHC Ligand Assay (**HLA-A∗11:01**)	SUDIPG33 (HLA-A∗11:01)
TP53	Arg175His	EVVR**H**CPHHER^a^	EVVRRCPHHER	HLA-A∗68:01	27.2	11	MHC Ligand Assay (HLA-A∗33:03)	QIMRFPW1 (HLA-A∗68:01)
TP53	Cys135Phe	YSPALNKMF**F**^a^	YSPALNKMFC	HLA-B∗15:17	37.4	10	MHC Ligand Assay (HLA-A∗24:02)	SKNBE2 (HLA-B∗15:17)
TP53	Tyr220Cys	VVP**C**EPPEV	VVPYEPPEV	HLA-C∗16:01	7.6	9	MHC Ligand Assay (HLA-A∗02:01) & T cell Assay	EW8 (HLA-C∗16:01)

Validated HLA restrictions matched with predicted binding of HLA allele were shown in bold, and the mutated residues were underlined.

a Peptides with weak evidence (single supporting study).

### Transcriptional profiling of human leukocyte antigen and antigen presentation genes in the Childhood Cancer Model Atlas cohort

**Figure 5:**
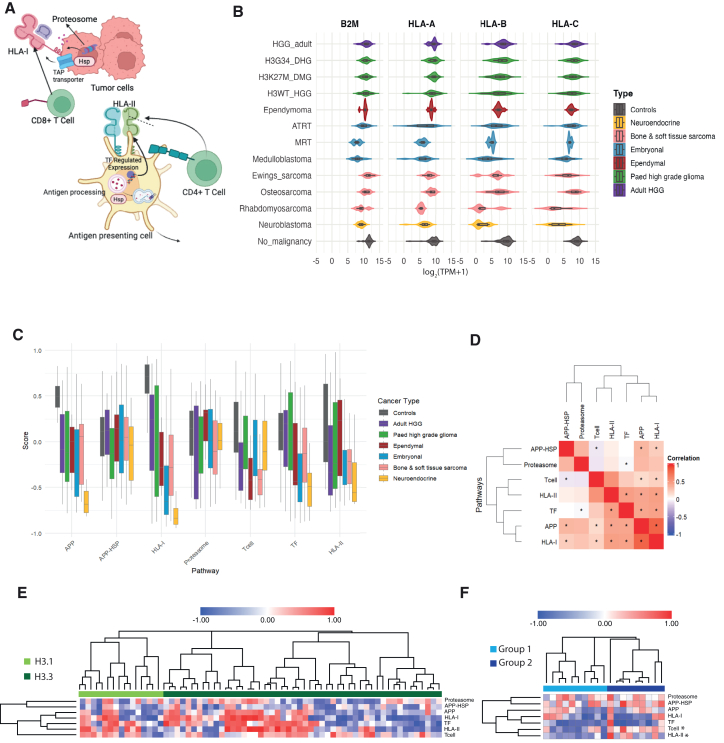
Transcriptional profiling of HLA and antigen presentation genes in the CCMA Cohort (A) Schematic plot shows transcriptional profiles of cellular pathways and key regulators in HLA and antigen presentation. (B) Violin plots show the expression of individual HLA genes (*HLA-A*, *HLA-B*, and *HLA-C*) across CCMA cancer types. No significant differences were identified using one-way ANOVA. (C) Aggregated pathway boxplot categorized by cancer types, highlighting differential immune-related pathway activity. (D) Correlation plot illustrates significant relationships among APP, HLA class I, HLA class II, and proteasome pathways. (E and F) Heatmaps showing pathway activity scores for key immune-related and proteasome pathways across distinct subtypes of H3K27M-DMG (E) and ATRT (F), namely DMG-H3.1K27M and DMG-H3.3K27M, Group 1 and Group 2 ATRT. Each row represents a specific pathway, and each column corresponds to an individual tumor sample. Color scheme indicates relative expression levels, red indicates increased activity, white neutral expression, and blue downregulation. Hierarchical clustering, using Ward’s method with Euclidean distance. ∗ Denotes FDR-adjusted *p* < 0.05 assessed using a two-tailed Student’s t test or Wilcoxon rank-sum test, with multiple testing correction via the Benjamini-Hochberg procedure. See also Figures S3 and S4, Tables S4 and S6.

HLA class I and II molecules present epitopes to CD8^+^ and CD4^+^ T cells, respectively (Figure 5A). The HLA class I complex comprises a heavy chain (encoded by *HLA-A, HLA-B, or HLA-C*) and β2-microglobulin (B2M). The expression of these components is known to vary among pediatric tumor types.^48^ Therefore, we first assessed the expression level (log_2_[TPM+1]) of individual HLA class I genes (*HLA-A*, *HLA-B*, and *HLA-C*) and *B2M* to uncover their intrinsic capacity for immune engagement (Figure 5B). High-grade gliomas showed the highest HLA class I expression, with median *HLA-A* between 8.8 and 9.5 and *HLA-C* between 7.7 and 8.4 across H3G34-DHG, H3K27M-DMG, H3WT-HGG, and adult HGG, consistent with stronger antigen presentation. By contrast, neuroblastoma had low levels (*HLA-A* 6.5; *HLA-B* 1.0; *HLA-C* 3.4). Embryonal tumors (ATRT, ETMR, MRT, and medulloblastoma) were with similarly low expression levels, with medians spanning 6.0–7.2 for *HLA-A*, 3.9–6.5 for *HLA-B*, and 5.9–8.0 for *HLA-C*, indicating limited HLA class I-mediated immune visibility. Additionally, given the limited immune stimuli in the tissue culture environment, HLA class II expression (overall median value 0.1), such as HLA-DR and HLA-DQ, was generally lower in the CCMA cohort compared to HLA class I genes (overall median value 8.25; Figure S3A).

In addition, we assessed the activity of key pathways that regulate antigen presentation and immune response, which are critical for understanding tumor antigen presentation and identifying vulnerabilities in immune-evasive tumors (Figure 5A). The proteasome degrades intracellular proteins into peptides, which are then processed and loaded onto MHC class I molecules via the antigen processing and presentation (APP) pathway. APP-HSP, involving HSP70 and HSP90 heat shock proteins, facilitates peptide stabilization and transport, enhancing immune recognition. Key transcription factors (TFs), such as *CREB1*, *CIITA*, and *ICAM1*, regulate the expression of immune-related genes, including those involved in antigen processing and HLA class I/II expression, thereby influencing tumor antigen presentation. Additionally, T cell related pathways modulate activation and differentiation, further shaping the immune response. The coordinated expression of HLA class I/II directly impacted tumor immune recognition. In CCMA, neuroblastoma exhibited the lowest antigen presentation levels, consistent with the low expression of HLA class I genes (Figures 5C and S3B). Interestingly, tumor-associated cells (controls) within the tumor microenvironment, such as tumor fibroblasts, exhibited stronger expression of APP, APP-HSP, HLA class I and II, suggesting that the microenvironment may play a compensatory role in antigen presentation. Notably, HLA class I gene sets showed the most variable expression patterns, reflecting fundamental differences in how tumors present peptides on the cell surface. Furthermore, we conducted correlation analyses (Figure 5E) to reveal potential co-regulation of the pathways. These analyses revealed a significant positive correlation between APP and proteasome activity (R = 0.45, *p*-value <0.01), indicating that these processes may be tightly regulated together. APP activity showed strong associations not only with the proteasome but also with APP-HSP, HLA class I and II expression, suggesting coordinated regulation within the antigen processing and presentation machinery.

CCMA holds the largest collection of high-grade brain cancer cell models, providing molecular diversity that enables deeper insights into the biology of pediatric CNS cancer subtypes. To address whether antigen presentation patterns correlate with subtype-specificity, we compared pediatric DMG with H3.3K27M (encoded by *H3-3A*, *n* = 50) and H3.1K27M (encoded by *H3-C*2 and *H3-C1*, *n* = 15). No significant differences were identified between H3.3 and H3.1 DMG models (*n* = 65; Figures 5E and S4A), with substantial variation identified within H3.3 subgroups. These results indicate that antigen presentation has a weak association with the specific H3K27M mutation. In contrast, ATRT Groups 1 and 2 exhibited clear differences in pathway activity (Figure 5F). For example, ATRT-Group 2 displayed significantly higher expression of HLA II pathway activity with a fold change of 2.2 (*p* = 0.026, FDR = 0.093), suggesting a greater degree of immune recognition and potential antigen presentation compared to Group 1 (Figures 5F and S4B). T-cell signaling pathways also exhibited a clear subtype-specific trend, with ATRT-Group 1 showing notably lower activation (*p* = 0.010, FDR = 0.081) (Table S4). This difference in T-cell pathway activity indicates a more immunosuppressed microenvironment in Group 1 relative to Group 2, pointing to distinct immunological profiles that may influence therapeutic responses.

### Childhood Cancer Model Atlas models maintain comparable human leukocyte antigen class I expression levels to matched tumors

A central question is whether the cancer type specific hierarchy of HLA class I gene (*HLA-A*, *HLA-B*, and *HLA-C*) expression observed in the CCMA cohort is preserved in primary tumors. Building upon the immunogenic profiles established in the CCMA cell lines, we next compared their transcriptomic data to pediatric primary tumors and additional pediatric cancer-derived cell lines from the Open Pediatric Cancer (OpenPedCan) initiative. This comprehensive dataset comprises 335 solid tumor samples and 155 cell lines covering more than 20 pediatric cancer types, providing a robust framework for assessing whether *in vitro* models faithfully recapitulate *in vivo* tumor biology.^49^^,^^50^

**Figure 6:**
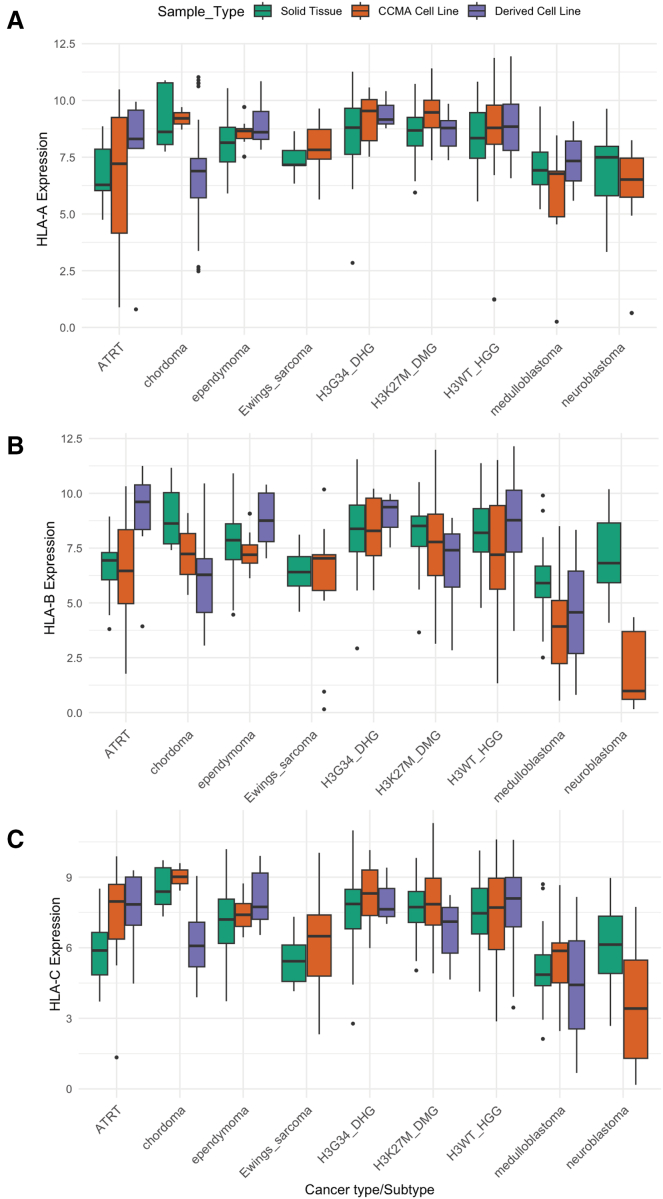
HLA class I gene expression across pediatric cancer subtypes in different sample types (A–C) Boxplots display log2-transformed transcript per million (TPM) expression values of *HLA-A* (A), *HLA-B* (B), and *HLA-C* (C) across various pediatric cancer subtypes in solid tumor samples (green) and derived cell lines (purple) from OpenPedCan, and CCMA cell lines (orange) from our analysis. Statistical significance was determined by a two-way ANOVA test (*p* < 0.001). Data are presented as boxplots showing the median (center line), interquartile range (box), and whiskers extending to 1.5 × the interquartile range; individual points represent outliers. See also Figures S5 and S6 and Table S5.

A two-way ANOVA test (*p* < 0.001) (Figure S5; Table S5) confirmed significant differences between sample types, i.e., cell lines and tumor tissues, but the preservation of cancer-specific patterns underscores the intrinsic nature of these immunogenic profiles (Figure 6). Although immune cell-mediated suppression is absent *in vitro*, many tumor-intrinsic immune escape mechanisms, such as altered HLA class I expression are retained. As a result, cell lines may still exhibit comparable absolute expression of HLA class I genes compared to tumor tissues.^51^ Indeed, we found that the relative ordering of tumor types largely remains comparable, with ependymomas displaying the highest HLA class I expression, whereas neuroblastoma, ATRT, and medulloblastoma formed a lower-expression group (Figure 6). In contrast to HLA class I, HLA class II genes (e.g., HLA-DR, HLA-DQ, and HLA-DP) were significantly more highly expressed in primary tumors than in any of the cell line cohorts, due to lack of immune stimuli in the tissue culture system (CCMA or OpenPedCan; Figures 6A–6C and S4). This observed upregulation of HLA class II gene expression in primary tumors, compared to cell line cohorts, underscores the pivotal role of the tumor microenvironment-encompassing cytokines, immune infiltrates, and other regulatory signals-in modulating these immune-related processes.

Beyond individual HLA genes, we examined whether the proteasome and antigen processing and presentation (APP) pathways exhibit cancer type/subtype specific patterns in both primary tumors and cell lines (Figure S6A). Despite the presence of cytokine-driven modulation *in vivo*, key pathway trends identified in CCMA-derived cell lines were largely mirrored in solid tumor samples. Notably, neuroblastoma exhibited significantly low APP and HLA class I activity, despite significantly elevated proteasome and APP-HSP activity (Figure S6A). Although neuroblastoma tumors have generally been reported to express low levels of HLA, recent studies have shown that HLA class I expression is higher in heterogeneous neuroblastomas with elevated mesenchymal markers.^52^^,^^53^ Consistent with these findings, our neuroblastoma models are enriched for adrenergic gene signatures and show low expression of mesenchymal signatures (Figure S6B). By contrast, ATRT remained consistently low across sample types, reflecting a more immune-evasive phenotype. These highly significant two-way ANOVA findings (Figure S6A) indicate that while many core antigen-processing and presentation mechanisms are conserved, their relative activity can shift depending on tumor subtype and *in vitro* versus *in vivo* contexts.

## Discussion

While extensive immunogenic characterizations and HLA typing have been reported for large cancer cell line cohorts such as CCLE,^54^ pediatric cancer models have lagged behind these efforts. HLA typing is essential for preclinical research, particularly in advancing immune-based therapies, including personalized vaccines, TCR-T therapies, and other immunotherapeutic approaches. In this study, we compiled a comprehensive catalog of HLA class I types and neoepitopes for nearly 300 high-grade pediatric cancer cell lines in the CCMA, which includes both widely used and newly established models. This dataset represents the largest collection of RNA-seq data for pediatric high-grade cancer cell models, particularly for CNS tumors. This publicly available resource serves as a centralized dataset, enabling researchers to accelerate hypothesis testing and streamline preclinical investigations. We analyzed the immunogenic landscape, assessing HLA class I and HLA class II expression across different cancer types and subtypes.

The CCMA demonstrated broad coverage of HLA alleles and supertypes, including prevalent alleles across ethnicities and rare alleles with dual specificity supertypes (“A01 A03” and “A01 A24”). Previous studies have investigated associations between HLA supertypes and clinical outcomes in adult cancers. The B44 supertype was reported to be associated with extended survival in patients with melanoma with ICB therapy, whereas the B62 supertype was associated with poor outcome.^32^ In pediatric cancers, one study reported HLA class I allelic and supertype diversity in patient-derived xenograft (PDX) models of varied pediatric cancer types.^55^ Similar to our results, their PDX panel captured most of the HLA class I alleles observed in pediatric solid tumor patient cohorts. Additionally, specific HLA class II alleles were found to be associated with pediatric cancer risk. HLA-DRB1∗03:01 was associated with osteosarcoma susceptibility, and HLA-DQB1∗05:02 was associated with a higher risk of developing neuroblastoma. Notably, no HLA class I alleles showed a significant association with pediatric cancer risk in both studies.^56^^,^^57^ Overall, our atlas captures substantial molecular and HLA allelic diversity across pediatric cancer models, offering a broad representation of HLA allotypes and supertypes to inform study design and neoantigen discovery.

The CCMA encompasses models with varying degrees of HLA class I homozygosity, with 23 models featuring homozygous HLA types across all HLA class I genes. We suspect the high homozygosity frequency stems from HLA LOH through aberrations to chromosome 6 or focal deletions of the HLA locus. Furthermore, HLA class I allele-specific transcriptional downregulation (MAF <0.25) was detected in 79 models (Figure 3B). Both observations may contribute to restricted peptide presentation and are, therefore, important factors to consider when selecting cancer models for testing hypotheses. Disrupted HLA class I expression helps tumors evade immune detection, but complete loss increases their vulnerability to NK cell surveillance due to the absence of self-antigen presentation.^58^ Therefore, partial HLA class I down-regulation may enable tumors to escape both T cell and NK surveillance.^59^ HLA LOH and ASE loss have been frequently observed across many adult cancer types, including pancreatic cancer,^60^ bladder cancer,^61^ and lung cancer.^62^ HLA LOH has also been reported in pediatric cancer.^63^ Consistent with our findings, HLA homozygosity were more frequently observed in sarcomas, particularly osteosarcomas,^63^ which is likely associated with the known genomic instability. HLA class I homozygous CCMA models provide a valuable resource to elucidate the consequences of a reduced epitope repertoire, whereas cell line models exhibiting reversible ASE loss enable the investigation of the therapeutic benefit in recovering the expression of the lost HLA allele with interferons or inhibitors.^64^^,^^65^ However, extensive passaging of cell lines can introduce excess genetic stress and contribute to LOH.^66^ Furthermore, we acknowledge that the observation of homozygous HLA alleles in our data could be attributed to germline HLA homozygosity.

*In silico* prediction of neoepitopes from CCMA models identified several public neoantigens derived from recurrent cancer driver mutations (Tables 1 and S3.5). We found H3.3K27M derived neoepitopes predicted to bind to various HLA alleles in patient models. The H3.3K27M mutation is highly recurrent in pHGG. The HLA-A∗02:01 restricted neoepitope H3.3K27M_26-35_ (RMSAPSTGGV) has shown promise in T cell recognition^41^^,^^42^ and its peptide vaccine resulted in increased survival in pediatric patients with DMG.^43^ However, recent evidence suggests the T cell recognition of the H3.3K27M_26-35_ neoepitope cannot be replicated and is not endogenously presented by HLA-A∗02:01 in DMG models.^44^^,^^47^ Similarly, the HLA-A∗03:01-restricted H3.3K27M_26–36_ neoepitope, which was also predicted by our pipeline, has been shown to be presented by HLA-A∗03:01^+^ HEK293T cells transfected with H3.3K27M. However, to this date, it has not been naturally detected in HLA-A∗03:01^+^ DMG cell lines.^47^ Despite this, the H3.3K27M mutation remains a prime neoantigen target due to its recurrence and broad applicability to patients with DMG. As shown by the INTERCEPT H3 trial, which demonstrated the endogenous presentation of a long H3.3K27M_14-40_ peptide by HLA class II and CD4^+^ T cell mediated immune responses.^67^^,^^68^ This highlights the great potential of HLA class II restricted antigen presentation in cancer immunotherapies. One putative H3.3K27M neoepitope predicted from our study was the H3.3K27M_22-30_ (ATKAARMSA) peptide, which was predicted to bind to HLA-A∗30:01 with high affinity and high likelihood of presentation (percentile rank of 0.02 by NetMHCpanEL) in five models (Table S3.1). Notably, this H3.3K27M_22-30_ peptide was also predicted from the study by Chang et al., 2017 with NetMHCcons v1.1.^23^ The H3.3K27M_22-30_ peptide may be a promising candidate for immunogenicity validation. The *ALK* F1174L mutation is also a recurrent driver mutation observed in primary neuroblastomas.^69^^,^^70^^,^^71^ Neoepitopes derived from this mutation were predicted across five neuroblastoma models. Among these, a 9-mer (MEALIISKL) was commonly predicted by Chang et al., 2017^23^ (Table S3.4). Besides the 9-mer peptide, we also predicted epitopes with different lengths (11-mer: FLMEALIISKL; 8-mer: EALIISKL) from the same mutation. These *ALK* F1174L associated neoepitopes are predicted to bind to a range of HLA alleles (HLA-A∗02:01; HLA-B∗08:01; HLA-B∗49:01; HLA-B∗51:01), which may suggest their broad applicability and potential for immunotherapy development.

Splicing and fusion-derived variants are of particular interest in pediatric cancers due to the prominent prevalence of fusion-driven tumorigenesis^72^ and the emerging recognition of splicing alterations as key contributors to tumor biology,^73^ making them attractive candidates for neoantigen discovery and immunotherapeutic targeting. The neoantigen candidates arising from the *PSPH*: c.275+1del splice variant are predicted to be likely benign, but this mutation has been observed as a somatic mutation in several patients with tumor (cBioPortal), suggesting its potential relevance in tumor cells. The *EWSR1*::*FLI1* fusion is a hallmark of Ewing sarcomas.^74^ A total of 24 predicted neoepitopes from different fusion rearrangements were observed in nine Ewing sarcoma models. Interestingly, *EWSR1*::*FLI1* epitopes are predicted to bind to *HLA-C* predominantly (23 out of 24). Compared to the results of Chang et al., 2017,^23^ we observed epitopes with highly similar sequences with 1–2 mismatches. Consistent with the prediction data of PCGP, *EWSR1*::*FLI1* fusions were capable of generating multiple predicted effective epitopes within a single model.

*TP53* mutations and predicted neoepitopes were also frequently found in CCMA models. The TP53Y220C_217-225_ (VVPCEPPEV) neoepitope has been well characterized for immunogenicity from multiple studies^75^^,^^76^ (Tables 1 and S3.5). Notably, CD8^+^ T cell responses to the TP53R248W_240-249_ neoepitope restricted to HLA-A∗68:01 were observed in patients with epithelial cancers.^75^^,^^77^ Although the TP53 R248W_240-249_ (SSCMGGMNWR) neoepitope was not predicted from our data, we observed predicted binding and presentation (percentile rank of 0.49 by NetMHCpanEL) from the TP53 R248Q_240-249_ (SSCMGGMNQR) peptide with HLA-A∗11:01 in the SUDIPG33 model (Tables 1 and S3.5). Interestingly, the well-studied TP53 R175H_168-176_ neoepitope^78^ was not identified from our predictions. As previously reported, the TP53 R175H_168-176_ peptide performs poorly in computational binding prediction.^78^^,^^79^ While we found the TP53 R175H mutation present in four HLA-A∗02:01^+^ cell lines, the TP53 R175H_168-176_ neoepitope was predicted by NetMHCpan 4.1 as a non-binder to HLA-A∗02:01 at 4543.17 nM (rank 7.5%).

In addition, evidence suggests neoepitopes with low binding affinity and avidity can elicit potent antitumor immunity.^80^^,^^81^ The demonstrable strengths and benefits of current neoepitope prediction approaches provide valuable utility and enable candidate discovery and prioritization for downstream experimental validation. The CCMA provides a valuable platform to investigate peptide processing and presentation for novel HLA allele and neoepitope combinations.

CCMA cell lines largely preserved cancer type specific HLA class I expression patterns, supporting their relevance to immuno-oncology research. However, primary tumors exhibited higher HLA class II expression, likely driven by immune-stimulating factors such as cytokines (including interferons) present *in vivo*. These findings suggest that tumor models can effectively capture intrinsic antigen presentation dynamics but may require an additional microenvironmental context to fully replicate immune interactions.

Our comprehensive evaluation of transcriptional signatures of antigen processing and presentation pathways in the CCMA cohort underscores the varying degrees of immune engagement across pediatric tumor types and subtypes and highlights the influence of both intrinsic tumor characteristics and extrinsic microenvironmental factors. High-grade gliomas (e.g., H3K27M-DMG, H3G34-DHG) exhibited elevated HLA class I (*HLA-A*, *HLA-B*, and *HLA-C*) expression, facilitating stronger immune recognition, while embryonal tumors (e.g., medulloblastoma, MRT) displayed significantly lower levels, suggesting an immune-cold phenotype with reduced antigen presentation potential. Cellular pathway analysis reinforced these findings, revealing that embryonal tumors consistently showed lower proteasome and APP activity, indicative of an intrinsic tendency toward immune evasion. These findings align with earlier reports demonstrating that HLA-I-mediated antigen presentation can significantly dictate tumor immune recognition to cytotoxic T cells.^82^^,^^83^ Further analysis revealed co-regulation between proteasome and APP pathways, particularly emphasizing the role of heat shock proteins (e.g., HSP70, HSP90) in stabilizing peptides and facilitating MHC class I loading.^84^ Notably, subgroup-level heterogeneity was evident, with ATRT Group 2 (MYC-high) demonstrating higher HLA class II expression and greater T cell signaling activity than Group 1, underscoring molecular subgroup-specific differences in immune engagement.^85^ While this distinction indicates that Group 2 tumors may have a greater capacity for immune engagement, ATRT as a whole remains characterized by low baseline APP activities and immune evasion mechanisms.

Therapeutically, our findings suggest that tumors with intact antigen-processing and presentation machinery (e.g., high-grade gliomas) may be more amenable to immune-based interventions, though such strategies should be carefully balanced to account for the potential upregulation of immunosuppressive pathways (e.g., PD-L1 via IFNγ signaling). Conversely, immune-cold tumors such as medulloblastoma and ATRT may require alternative approaches, such as epigenetic modulators to restore MHC expression or HLA-independent adoptive cellular therapies (e.g., CAR-T or NK cell-based treatments) that circumvent classical antigen presentation barriers. Recognizing these tumor and subgroup-specific immunogenic signatures could refine immunotherapeutic approaches, enabling more targeted and effective treatments for pediatric malignancies.

The characterization of immunogenic features reported in this study carries significant clinical implications, especially given treatment-related toxicities observed in high-risk pediatric cancers. This cohort prominently features CNS tumors, which remain the leading cause of disease-related mortality, while also including rare and under-characterized pediatric cancer types. Our resource paves the way for novel immunotherapeutic strategies aimed at pediatric cancers associated with poor survival outcomes and severe long-term side effects. Although the current study primarily emphasizes HLA class I typing and neoepitope prediction in high-risk tumors, it also provides a versatile platform that can be expanded to incorporate additional molecular characteristics relevant to advancing immunotherapy development.

### Limitations of the study

While this comprehensive analysis advances our understanding of the landscape of HLA type, HLA expression, and neoepitope candidates in pediatric cancer, several limitations warrant consideration. First, due to the lack of paired germline samples and the complexity of the polymorphic HLA region, conventional copy number tools applied to tumor-only WGS data may be less reliable in detecting allele-specific or focal LOH events at the HLA locus. While large-scale deletions are often detectable, the influence of HLA polymorphism on the resolution of focal LOH remains a possible source of analytical bias. Second, neoepitope predictions are based on computational algorithms and are subject to both false positives and false negatives, underscoring the need for experimental validation. Third, the prolonged passaging of cell lines across different laboratories may introduce genetic drift and varying selective pressures, including HLA LOH, potentially confounding interpretations of HLA homozygosity and ASE loss. The absence of the native tumor microenvironment in cell lines limits the extrapolation of these findings to *in vivo* contexts. Despite these limitations, the resulting atlas offers a valuable resource to support hypothesis generation and prioritization for downstream experimental and translational studies in pediatric cancer immunotherapy.

## Resource availability

### Lead contact

Further information and requests for resources and reagents should be directed to and will be fulfilled by the Lead Contact, Dr Claire Xin Sun (claire.sun@hudson.org.au).

### Materials availability

This article does not report novel material or reagents generated.

### Data and code availability

• Datasets are publicly available at and deposited at “CCMA primary multi-omics datasets,” Mendeley Data, V1, https://doi.org/10.17632/rnfs539pfw.1. HLA typing output from WGS and RNA sequencing are in Table S1.
• This article does not report original code.
• Any additional information required to reanalyze the data reported in this article is available from the lead contact upon request.

## Acknowledgments

Funding for the project is from the Medical Research Future Fund MRF2030828. This project is also supported by the Next Generation Precision Program, which is/was generously supported by the Children’s Cancer Foundation Australia, 10.13039/501100016057Robert Connor Dawes Foundation, and the Medical Research Future Fund (10.13039/501100000925NHMRC Project 2007620). C.S. is funded by Australia Victoria Cancer Agency ECRF22006. We thank over 30 contributing sites for providing cell lines and/or tissues from which cell lines were generated. A full list of cell line contributors is provided in supplemental information. This work was supported by Monash eResearch capabilities, including data storage and high-performance computing resource MASSIVE M3.

We wish to acknowledge the generosity of the following collaborators who contributed the following cell line models. This article and research was made possible in part due to The Children’s Brain Tumor Network (CBTN). The following cell lines were provided by CBTN: 7316-2141 S, 7316-2187 S, 7316-4149 S (Occipital), 7316-4149 S (Cerebellum), 7316-1763 S, 7316-195 S, 7316-3058 A, 7316-3058 S, 7316-388 A, 7316-6349 S, 7316-6475 S1, 7316-24 S, 7316-5335 S2, 7316-5335 S1, 7316-913 S, 7316-1746 A, 7316-1763 A, 7316-1781 A, 7316-2141 A, 7316-2151 S, 7316-2176 A, 7316-2186 A, 7316-2187 A, 7316-2189 A, 7316-2582 A, 7316-3045 A, 7316-445 A, 7316-85 A, 7316-913 A, 7316-4062 S, 73161769S, 731610374S1, 7316406S1, 7316-1746-S7316-158-S, 7316-4509-S, 7316-4906-S, 7316-5317-S14 and 73168121S2. This article would not have been possible without the support of the Zero Childhood Cancer Personalised Medicine Program (ZERO) at Children’s Cancer Institute Australia (CCIA). Zero provided the following cell lines: - P001401, P002306, P003302, P005306, P005401, P007401, RA002, RA055, P001003, P009802, P008401, P011601, P001001, P001802, P003201, P000901 and P006507. We acknowledge Dr. Anat Erdreich-Epstein of Children’s Hospital Los Angeles and Grayson’s Gift Foundation as the provider of the following cell lines: CHLA-02, CHLA-04, CHLA-05, CHLA-06. We acknowledge Dr. Javad Nazarian of Children’s National Hospital as the provider of cell lines CNMC 760 XD and CNMC-D 874. The article and research were made possible in part due to the contribution of CHLA-200, CHLA-259, CHLA-266, BT-12 from the Children’s Oncology Group and the COG Biopathology Center (BPC). The Children’s Hospital of Philadelphia is responsible for administrative oversight and support of COG-conducted clinical trials under a grant provided by 10.13039/100000054the National Cancer Institute (NCI). The Children’s Oncology Group Cell Culture/Xenograft Repository provided material for a part of this research. We would like to thank Dr. Peter Houghton for providing us with access to the cell line BT-16. We acknowledge Dayton Children’s Hospital for its contribution in providing cell line 168MNU1210. We acknowledge Duke University as the source of cell lines D425 and D283. The KNS-42 cell line was supplied by the JCRB Cell Bank (Osaka, Japan) as catalog number IFO50356, and was purchased from CellBank Australia (Westmead, NSW, Australia). The cell line has been described before: Characteristics of an established human glioma cell line, KNS-42. Takeshita I,Takaki T,Kuramitsu M,Nagasaka S,Machi T,Ogawa H,Egami H,Mannoji H,Fukui M, Kitamura K Neurol Med Chir (Tokyo). 1987 Jul;27(7):581-7. We acknowledge the Fred Hutchinson Cancer Research Center as the provider of the following cell lines: ATRT-310FHTC, ATRT-311FHTC, GBM-511FHTC, Med-114FHTC, PBT-04FHTC, PBT-05FHTC, Med-411FHTC, EPD210FHTC, GBM110FHTC, PBT22FHTC and PBT29FHTC, and PBT27FHTC. The cell lines B143, G401, HEK-293, HOS, MG63, OHS, OS25, RN1, SAOS2, SJSA-1, U118-MG, U87-MG, A-172, D341, and DAOY were obtained from the American Type Culture Collection (ATCC). We acknowledge Dr. Keith L. Ligon from Dana-Farber Cancer Institute as the source and Dr. Nada Jabado of McGill University as the provider of the following cell line: BT245 par (H3.3 K27M parental), and BT237. We acknowledge Dr. Maria Vinci of Bambino Gesù Children’s Hospital as the provider of the cell lines OPBG-DIPG-004-2D, OPBG-GBM-001, OPBGDIPG002, OPBGDIPG019A, OPBGDIPG019B, OPBGGBM004, and OPBGGBM005. We acknowledge Dr. Jeffrey Rubens and Dr. Eric Raabe of John Hopkins University as providers of the following cell lines: JHH-DIPG-1 (JHH-1-J), JHH-DIPG-2J, and BT-37. Tumor samples and coded data were supplied by the Children’s Cancer Center Tissue Bank at the Murdoch Children’s Research Institute and The Royal Children’s Hospital (www.mcri.edu.au/childrenscancercentretissuebank). Establishment and running of the Children’s Cancer Center Tissue Bank is made possible through generous support by Cancer In Kids @ RCH (www.cika.org.au), Leukemia Auxiliary at RCH (LARCH), the Murdoch Children’s Research Institute, and The Royal Children’s 10.13039/501100022917Hospital Foundation. We also acknowledge The Royal Children’s Hospital as the provider of fresh tissue sample RCH4065, RCH10072, RCH9412, RCH9689, RCH10508, RCH10654, RCH10880, RCH10826, RCH6417 and RCH11001. We acknowledge Dr. Terry Johns as creator of the following cell lines: GBM39, OSL1 and GBM6. We acknowledge Monash Health and Monash Children’s Hospital as the provider of the following fresh tissue samples: PB17SH009 (BSC-ATRT), PB19SH009, PB19SH058, PPMP002-250919, PPMP020-280220-GG, PB18SH035, PPMP007-110520, PPMP007-181019, PPMP011-261119 CHC, PPMP032-101120-CPC, PPMP046 220121, PPMP052-040821 Left Bone Marrow and PPMP052-040821 Right Bone Marrow, PB22SH109, PB22SH145, PPMP002-071122, PPMP059-130322, PB23SH002, PB23SH036, PB23SH031, PB23SH090, PB24SH017S1, PB24SH017S2, PPMP017-210220-MED, PPMP018040320-ESARC, PPMP022-120320-ANGIO, PPMP024-030720-NBL, PPMP026-310720-RSARC, PPM-058-140222, PB18-SH-020, PB18-SH-036, PB18-SH-074, PB19-SH-016 and PPMP048-070820. We acknowledge Children’s Hospital Los Angeles as the provider of cell line STM91-01. We acknowledge Dr. Jae Cho and Boston Children’s Hospital for their part as the provider of cell line JC ATRT. We acknowledge Professor Bryan Day of QIMR Berghofer and Dr. Brett Stringer of Flinders University for their part as the providers of the following cell lines: QIMR-SJH-1, QIMR-FPW-1, QIMR-HW-1, QIMR-MMK-1, QIMR-RN-, QIMR-BAH1, QIMR-WK1, PR1.1, RR2, QIMRPB1, and QIMRRKI1. We acknowledge Dr. Angel Carcaboso of Sant Joan de Déu Barcelona Hospital for their part as the provider of cell lines HSJD-GBM-002 and HSJD-DIPG-007. We acknowledge Dr. Michelle Monje of 10.13039/100005492Stanford University, and 10.13039/100017296Swifty Foundation, and 10.13039/100018101the McKenna Claire Foundation (grant to M. M.) for their part as the provider of following cell lines: SU-DIPG 13, SU-DIPG 17, SU-DIPG 19, SU-DIPG 21, SU-DIPG 24, SU-DIPG 25, SU-DIPG 27, SU-DIPG 29, SU-DIPG 33, SU-DIPG 35, SU-DIPG 36, SU-DIPG 38, SU-DIPG 4, SU-DIPG 6, SUPSCG1 (SU-pSCG-1), and SUPSCGBM2 (SU-pcGBM2). We acknowledge the Institute of Cancer Research (ICR) for their part as the source of material used in this research. We are thankful for the support of Prof. Chris Jones and Dr. Diana Carvalho for their contribution as provider of following cell lines: ICR-B181-2D, ICR-B169-2D, ICR-B184-2D, ICR-B194-2D, ICR-B301-2D, ICR-CXJ-001-3D, ICR-CXJ-046, DUB-D003-2D, ICRCXJ0082D, ICRCXJ0083D, ICRCXJ0153D, ICRCXJ0163D, ICRCXJ0172D, ICRCXJ0242D, ICRCXJ026B2D, ICRCXJ0302D, ICRCXJ0622D, ICRCXJ0642D, ICRCXJ0742D and ICRCXJ0762D. We acknowledge Dr. Alejandro Sweet-Cordero of the University of California, San Francisco for his contribution as provider of cell lines OS052, OS186, OS526, and OS384. We acknowledge Stanford University as the creator and Dr. Siddhartha Mitra from University of Colorado for their contribution as providers of cell line SU_13808 (ATRT 13808). We acknowledge Dr. Amos Loh of Kandang Kerbau (KK) Women’s and Children’s Hospital and 10.13039/100020105VIVA Foundation for Children with Cancer (grant to A.H.P. Loh) as the provider of the following cell lines: HGG080318, HGG2909217, RHB231219, RHB140120, RHB130721, and RHB150623. We acknowledge Dr. Esther Hulleman of Princess Máxima Center for Pediatric Oncology as the provider of cell lines ATRT VU-397 SAK, VUMC A, VUMC-DIPG-11 and VUMC-DIPG-8, and VUMC-DIPG-10. We acknowledge Queensland Children’s Tumor Bank (QCTB) as the provider of tissue QCTB1593, QCTB1912, QCTB2427, and QCTB1641. We acknowledge the Global Harmonisation Consortium and collaborating institutions as the source of cell lines, Hopp Children’s Cancer Center (cell line I070_004), and St. Jude Children’s Research Hospital (cell line SJGBM2 (SJ-GBM2). We acknowledge MERCK as the provider of cell lines SF188, SF7761, and SF8628. We also acknowledge the University of California, San Francisco as the source of the above cell lines. We would like to thank Dr. Peter Houghton for providing us with access to the cell line SJSC. We also acknowledge St. Jude Children’s Research Hospital as the source of cell lines: SJDIPGX7C, SJHGGX6C, SJDIPGX37C, SJHGGX39C, SJDIPGX9C, SJHGGX42C, SJDIPGX29C, EW8, ES6LUC, ES1, RH41, and SJHGGX2C. Tumor samples and coded data were supplied by the Monash Children’s Cancer Biobank and the Monash Children’s Hospital. The Monash Children’s Cancer Biobank is generously supported by Bailey's Day, 10.13039/100001543Children's Cancer Foundation, 10.13039/501100015971Hudson Institute of Medical Research, the Monash Children’s Hospital Cancer Center, and Monash Health’s Pathology department. We acknowledge Dr. Dan Freed of the Institute of Pathology and the Chordoma Foundation as the provider of the UCH17 cell line. We acknowledge Dr. Maya Kansara of the Garvan Institute of Medical Research as the provider of the EP1NS cell line. We acknowledge the German Cancer Research Center as the provider of cell lines HDMB-03, TC205, TC71, and TC106. We acknowledge Leibniz Institute (DSMZ) GmbH as the provider of cell lines RDES, LAN1, A204, RH30, SKNMC, CHP100, MHHES1, LAN5, SKES1, GIMEN, and CADOES1. We acknowledge Assoc. Prof. Andrew Morokoff of Royal Melbourne Hospital is the provider of the cell lines: JJ252D and JJ412D.

## Author contributions

The studies were conceived and designed by C.S. and R.F. Y.G., I.M., and C.S. conducted computational analysis and wrote the article. V.A. conducted the analysis of HLA calling using Optitype. D.S. conducted pVACfuse analysis. I.W. contributed to analysis design and writing. T.S. and P.F. provided NGS HLA typing results.

## Declaration of interests

The authors declare no competing interests.

## STAR★Methods

### Key resources table

**Table:** 

REAGENT or RESOURCE	SOURCE	IDENTIFIER
**Deposited data**		

CCMA primary multi-omics datasets	Mendeley Data	https://doi.org/10.17632/rnfs539pfw.1
CCMA raw sequencing (WGS & RNA-seq)	European Genome-phenome Archive	EGAS00001006320
OpenPedCan RNA-seq	Open Pediatric Cancer (OpenPedCan) Project v15	https://pedcbioportal.kidsfirstdrc.org/study/summary?id=openpedcan_v15

**Experimental models: Cell lines**		

Pediatric tumor tissue (multiple histologies) used to derive cell lines	CCMA and Contributing hospitals and collaborators (see Table S1)	“this paper”

**Software and algorithms**		

OptiType v1.3.4	Szolek et al.^5^	https://github.com/FRED-2/OptiType
pVACtools	Hundal et al.^25^	https://pvactools.readthedocs.io/en/latest/
VAtools v5.1.1	Broad Institute	https://github.com/griffithlab/VAtools
VEP r113 + plugins (Frameshift, Wildtype)	Ensembl	https://www.ensembl.org/info/docs/tools/vep
BWA-MEM v0.7.17	Heng Li	http://bio-bwa.sourceforge.net
GATK v4.4 (Best Practices)	Broad Institute	https://gatk.broadinstitute.org
FastQC v0.12	Babraham Bioinformatics	https://www.bioinformatics.babraham.ac.uk/projects/fastqc
Yara aligner v0.9	Köster & Rahmann	https://github.com/Seqan/seqan
GSVA R package v1.48	Bioconductor	https://bioconductor.org/packages/GSVA
R v4.4.0 + tidyverse, ComplexHeatmap, etc.	R Core Team	https://www.r-project.org
NetMHCpan-BA/EL v4.1	DTU Health Tech	http://www.cbs.dtu.dk/services/NetMHCpan
MHCflurry v2.1	OpenVax	https://github.com/openvax/mhcflurry
MHCnuggets-I v2.3	Johns Hopkins Univ.	https://github.com/KarchinLab/mhcnuggets
BigMHC-EL/IM v1.0	Stanford Univ.	https://github.com/sjjer/BIG-MHC
arcasHLA v0.6.0	Orenbuch et al. 22	https://github.com/RabadanLab/arcasHLA
Regtools	Cotto et al.^86^	https://regtools.readthedocs.io/en/latest/
NetMHCStabPan v1.0	DTU Health Tech	http://www.cbs.dtu.dk/services/NetMHCStabPan
NetChop 3.0	DTU Health Tech	http://www.cbs.dtu.dk/services/NetChop
nf-core Sarek pipeline (v3.4.4)	nf-core	https://github.com/nf-core/sarek
nf-core hlatyping pipeline (v2.0.0)	nf-core	https://github.com/nf-core/hlatyping
nf-core rnafusion (3.0.2)	nf-core	https://nf-co.re/rnafusion/3.0.2/

### Experimental model and study participant details

Ethics approval for this work was obtained from the relevant committees (NMA HREC Reference: HREC/17/MonH/323; Monash Health Reference: 17-0000-389A; additional HREC Reference: 33207). Written informed consent for all subjects was obtained from their legal guardians. Consent documentation and biospecimens were collected by the Monash Children’s Cancer Biobank (Monash Children’s Hospital, Clayton, VIC) and the Children’s Cancer Centre Biobank (Royal Children’s Hospital, Parkville, VIC). Where applicable, patient age and sex are provided in Table S1. For cell line generation, tissue was transported in DMEM/F12 supplemented with GlutaMAX and Antibiotic–Antimycotic and processed within three days post-surgery. Details of the newly generated and established cell lines in this study (n=287) can be found in Table S1 and https://vicpcc.org.au/dashboard. All experiments conform to the relevant regulatory standards.

### Method details

#### Cell line sequence data

Whole genome and RNA sequences for 287 (249 and 275 respectively) cell lines were obtained from the Childhood Cancer Model Atlas as described in Sun et al., 2023^22^ (n=174) and newly updated datasets (n=113).^87^^,^^88^^,^^89^ Briefly, primary cell lines were generated in-house from tumor tissue or obtained from collaborators. Cell lines were validated by identifying hallmark genetic attributes through WGS or STR profiling. WGS was performed on extracted genomic DNA (DNeasy Blood & Tissue Kit) with 150bp paired-end sequencing. A minimum of 30X coverage was achieved for all cell lines. Raw data was processed using the nf-core Sarek pipeline^90^^,^^91^ (version 3.4.4). Briefly, sequences were preprocessed with GATK4 Best Practices^92^ and mapped to the human reference genome GRCh38 with BWA-MEM.^93^ Variants were called with Mutect2^92^ and annotated with snpEff^94^ and Ensembl Variant Effect Predictor (VEP).^95^

#### NGS amplicon sequencing high-resolution HLA typing

High-resolution HLA typing with NGS amplicon sequencing was performed for 29 cell lines (23 pHGG; 5 Bone & soft tissue sarcoma; 1 neuroblastoma; 1 control). Samples were processed at the Victorian Transplantation and Immunogenetics Service (West Melbourne, Victoria, Australia) and PathWest Laboratory Medicine (Perth, Western Australia, Australia). Complete or partial 4-digit, 6-digit, or 8-digit classical class I and II HLA types were obtained. IMGT/HLA sequence database^96^^,^^97^ versions 3.38.0, 3.50.0.0, and 3.53.0.0 were used as the reference database.

#### RNA expression levels quantification

For RNA sequencing, RNA was extracted (RNeasy Kit), poly-A enriched, processed as a sequencing library, and sequenced with paired-end 150bp sequencing. The nextflow pipeline nf-core/rnaseq (v3.19.0)^98^ was used to process raw RNA-Seq data to count and transcripts-per-million (TPM) matrices. Within the pipeline, reads were trimmed using fastp^99^ and their quality assessed using FastQC (https://www.bioinformatics.babraham.ac.uk/projects/fastqc/). Reads were then aligned to Ensembl release 111 of the human genome using STAR^100^ followed by alignment-based quantification by salmon. TPM data was summarized across replicates using the mean, followed by log base 2 normalization of the mean TPM plus 1 (log_2_(TPM + 1)).

#### HLA typing inference

The nf-core hlatyping pipeline^101^ (version 2.0.0) was used to determine 4-digit class I HLA types for 287 unique cell lines. In brief, raw DNA and RNA sequences were processed with FastQC, mapped to the human reference genome GRCh38 with Yara,^102^ and HLA types were determined with Optitype.^24^ Default settings were used, with enumerations set to 3. IMGT/HLA release 3.14.0 was used as the reference HLA sequence.

Four-digit HLA types were determined for the classical class I genes (*HLA-A*, *HLA-B*, and *HLA-C*) with a decision-making tree (Figure 1B). High-resolution HLA types were reported when available, as they are considered the most accurate method, superseding the NGS sequence-based typing results generated by Optitype.

Manual curation was used to determine non-concurrent HLA types. Cases include a match in 2-digit but not the 4-digit resolution or a complete mismatch between WGS and RNA-seq inferred typing. The alternative solutions (solutions 1 and 2) were used to resolve ambiguous HLA types. Preference was given to high-confidence alleles predicted across all three solutions. The curated HLA types were reported with an ambiguity flag. Cell lines with homozygous HLA types for all three genes were also flagged.

After determining the definitive HLA types, HLA supertypes for genes A and B were assigned to each cell line. HLA supertypes were defined according to the classification by Sidney et al., 2008.^27^ Before assigning the supertypes, HLA types were converted to the new nomenclature based on HLA allele designations from April 1st, 2010. All represented HLA alleles in our dataset were covered by supertype classifications.

#### Accuracy evaluation

Overlapping results between NGS high-resolution typing and Optitype were used to validate and calculate the accuracy of our strategy. Typing accuracy and zygosity prediction accuracy were defined according to Szolek et al., 2014.^24^ Briefly, typing accuracy was based on the percentage of correctly predicted 2-digit or 4-digit alleles. Zygosity prediction accuracy was determined by the percentage of correctly predicted homozygous and heterozygous loci, disregarding the correctness of the typed allele.

#### HLA zygosity analysis

HLA allele-specific expression was quantified using *arcasHLA-quant*^19^ (version 0.6.0). Briefly, HLA genotypes inferred from WGS using OptiType were used to build customized references. RNA-seq reads mapping to chromosome 6 were extracted and aligned to the references to assess expression loss of HLA alleles.

#### Neoepitope prediction

Neoepitope candidates were identified using the pVACtools^25^ cancer immunotherapy pipeline, including pVACseq (version 4.4.1), pVACfuse (version 5.3.0), and pVACsplice (version 5.3.0). Cell line-specific HLA types and mutation data were prepared and preprocessed according to pipeline instructions. Mutations were filtered to exclude germline variants labelled by Mutect2. Variants passing the Mutect2 hard filter were retained. The hard filter tags include clustered-events, duplicate, multiallelic, base-qual, map-qual, fragment, position, panel-of-normals, normal-artifact, and contamination. Variants were then annotated using the VEP^95^ (release 113) with the Frameshift and Wildtype plugins. To reduce computational load, the “--pick” option was used to retain the most impactful transcript for each mutation.

A variant consequence filter was applied to retain variants in the coding region. Using sequence ontology (SO) terms, variants with a consequence matching any child terms of coding-sequence-variant (SO:0001580) were selected. The retained SO terms include, stop-gained, frameshift-variant, stop-lost, start-lost, inframe-insertion, in-frame-deletion, missense-variant, protein-altering-variant, incomplete-terminal-codon-variant, start-retained-variant, stop-retained-variant, synonymous-variant, and coding-sequence-variant.

Gene expression data was integrated using VAtools (version 5.1.1) to annotate variants with gene expression levels in TPM. Somatic variants were used as input for pVACseq. A separate input of phased variants combining somatic and germline variations was prepared to account for proximal variants. Briefly, Mutect2 hard-filtered somatic variants and variants with the germline tag were retained. The combined variants were annotated with VEP and filtered for coding variants as described. ReadBackedPhasing in GATK^92^ (Version 3.6) was used to generate the phased variant input from WGS reads for pVACseq.

The pVACfuse pipeline was executed with the same settings as above to predict neoantigens from fusion variants annotated by Arriba (v2.5.0), using outputs from the nf-core/rnafusion pipeline (v3.0.2). Only fusions that passed Arriba’s internal filters were used for neoantigen prediction. Similarly, the pVACsplice pipeline was executed using junction mutations identified by RegTools.^86^ RegTools integrates splice region mutations and RNA expression data (from .bam files) to extract aberrant splice junctions and the resulting spliced isoforms.

The pVACtools pipeline was executed to generate all possible 8, 9, 10, and 11-mer epitopes. Seven prediction algorithms were used to evaluate peptide-MHC binding affinity (NetMHCpanBA,^103^ MHCflurry,^104^ MHCnuggetsI^105^), elution (NetMHCpanEL,^103^ BigMHC_EL,^106^ MHCflurryEL^104^), and immunogenicity (BigMHC_IM^106^). All possible downstream analysis options were specified. Epitope cleavage site was predicted with NetChop C term 3.0,^107^ peptide stability was predicted with NetMHCStabPan,^108^ and reference proteome similarity was determined with the human reference proteome GRCh38.

Promising neoepitope candidates were filtered with default parameters from pVACseq, with the IC50 scoring metric set to lowest. The applied filters include mutant allele IC50 binding scores below 500 nM, tumor DNA coverage > 10, tumor DNA variant allele frequency > 0.25, gene expression > 1, transcript support level ≤ 1, and only the top epitope was reported for each variant.

#### Tumor data sources

Gene expression data for the tumor tissues and external cell lines were obtained from the OpenPedCan^50^ repositories, which provide comprehensive RNA-sequencing profiles of pediatric cancers. The dataset included samples from key pediatric cancer subtypes, including gliomas, sarcomas, embryonal tumors, neuroendocrine and ependymal tumors. A total of 489 samples representing 9 subtypes were included after meeting the following selection criteria: (i) high RNA-seq quality scores, (ii) sufficient cancer type representation (≥ 2 samples per type), and (iii) availability of complete metadata, including histological subtype and sample type (cell line vs. solid tissue).

#### Gene set categorization for antigen presentation analysis

Pathway activity was assessed using Gene Set Variation Analysis^109^ (GSVA) with the GSVA R package (v4.4.0). GSVA does not require predefined sample labels, making it particularly suitable for detecting subtle and continuous changes in gene set enrichment across diverse tumor subtypes. Pathway gene sets were curated from the Molecular Signatures Database^110^ (MSigDB) and from existing immuno-oncology literature (Table S6). Key pathways of interest included antigen processing and presentation (APP), HLA class I and II, proteasome function, APP-HSP (heat shock protein–related antigen processing), T-cell activation, and transcription factors involved in immune. Gene panels were curated to represent distinct functional modules. Genes were grouped based on their known biological roles as follows: (1) core APP machinery (e.g., *TAP1*, *TAP2*, *TAPBP*), (2) chaperones and ER-associated heat shock proteins (APP-HSP), (3) HLA class I genes including *B2M*, (4) HLA class II genes, (5) proteasomal subunits involved in peptide degradation, (6) T cell specific markers (e.g., *CD4*, *CD8A*, *CD8B*), and (7) transcriptional regulators of immune response. Using the log_2_(TPM+1) expression matrix, GSVA calculated per-sample enrichment scores for each pathway, enabling direct comparisons across cell lines and primary tissues.

#### Gene set analysis of gene-expression signatures

Previously reported gene-expression signatures, including the neuroblastoma mesenchymal (MES) and adrenergic (ADRN) subtypes previously reported,^52^^,^^53^ were characterised using the GSVA package as described above. Signature scores were calculated for each sample.

### Quantification and statistical analysis

All statistical analyses were conducted in R (v4.4.0). Differences in pathway enrichment among subtypes were assessed using the Kruskal-Wallis test, with Dunn’s test for post hoc pairwise comparisons and Bonferroni correction to account for multiple testing. When comparing gene expression levels-such as HLA class I genes-across both subtypes and sample types (cell lines vs. primary tumors), two-way ANOVA was applied. Post hoc tests (e.g., Tukey’s HSD) were then performed when significant main effects or interactions were detected.

Correlation analyses between pathways (e.g., proteasome activity vs. APP) were performed using Pearson’s correlation coefficient. p-values from multiple comparisons were adjusted using the Benjamini-Hochberg method, and correlations meeting the adjusted threshold (FDR < 0.05) were deemed significant.

#### Reproducibility and software

Key R packages included ggplot2, ComplexHeatmap, and gridExtra for data visualization, alongside the base hclust function for hierarchical clustering. All scripts, configurations, and documentation are available upon request or in the supplemental information, ensuring transparency and reproducibility of the computational analyses.

#### Data availability

All processed RNA-seq datasets from OpenPedCan are publicly accessible through their respective repositories. CCMA RNA and SNP data are publicly accessible through its data dashboard and data portal at vicpcc.org.au/dashboard. Raw sequencing data has been published and archived at European Genome Archive EGA: EGAS00001006320 and is available from the lead contact upon agreement of the data access policy. Detailed metadata, including histological classifications and RNA expression profiles for OpenPedCan study and additional technical sequencing metrics may be available through the corresponding analysis pipelines on the OpenPedCan GitHub repository. HLA typing raw output from WGS and RNA sequencing are at Mendeley Data: https://doi.org/10.17632/rnfs539pfw.1.

## Supplemental information

Document S1. Figures S1–S6 and Tables S4–S6

Table S1. HLA class I typing for CCMA cell line models, related to Figure 1 1.1. HLA class I typing determined by OptiType, supertypes, zygosity, and cell line metadata. 1.2 Detailed concordance status of 28 models with mismatched WGS and RNA-seq inferred HLA types.

Table S2. Accuracy of HLA typing and zygosity, related to Figures 2 and 3 2.1 HLA prediction accuracy validated with NGS high resolution typing from 24 models. 2.2 Homozygosity/heterozygosity detection accuracy of HLA.

Table S3. Predicted neoepitopes for CCMA cell line models, related to Figure 4 3.1 Predicted neoepitopes with IC50 < 500 nM binding affinity from missense, in-frame insertion/deletions, and frameshift mutations determined by the pVACseq pipeline. 3.2 Predicted neoepitopes with IC50 < 500 nM binding affinity from alternative splicing determined by the pVACsplice pipeline. 3.3 Predicted neoepitopes with IC50 < 500 nM binding affinity from fusion mutations determined by the pVACfuse pipeline. 3.4 Commonly predicted neoepitopes with the Pediatric Cancer Genome Project cohort by Chang et al., 2017 [S1]. 3.5 Commonly predicted neoepitopes with IEDB.
