# Supplementary material for: Resource: A compendium of HLA types and expression in pediatric cancer models
Source: iScience. 2025 Oct 28;28(11):113887. doi: 10.1016/j.isci.2025.113887 (PMC12651366; doi:10.1016/j.isci.2025.113887)
Supplement: Document S1. Figures S1–S6 and Tables S4–S6 [file mmc1.pdf]

iScience, Volume *28*

Supplemental information

Resource: A compendium of HLA types and expression in pediatric cancer models

Yiwen Guan, Ishika Mahajan, Vikesh Ajith, Dingyin Sun, Isaac Woodhouse, Tima Shamekhi, Pouya Faridi, Ron Firestein, and Claire Xin Sun

Supplementary figure legend

Figure S1. HLA typing accuracy assessment for CCMA cohort. Related to Figure 2 and 3. (A) Stacked pie charts showing 2-digit and 4-digit concordance rate between mismatched cell line models. A total of 28 models with 168 HLA alleles were assessed. (B) Bar chart showing the accuracy of predicted HLA types. The percentage of match and mismatch HLA calls was inferred from comparing Optitype with high resolution typing. (C) Bar chart showing the accuracy of HLA zygosity prediction. The percentage of true positive (TP), true negative (TN), false positive (FP), and false negative (FN) classifications was inferred from comparing Optitype with high resolution typing.

Figure S2. Distribution of predicted neoepitopes across top 10 common HLA allotypes. Related to Figure 4. The distribution of 3,393 predicted neoepitopes among the top 10 most common HLA allotypes. Neoepitopes were passed all binding and elution model filters.

Figure S3. HLA gene expression profiles in the CCMA cohort. Related to Figure 5. (A) Box plot comparing HLA class I (HLA-I (blue): HLA-A, HLA-B, HLA-C) and HLA class II (HLA-II (red): HLA-DPA1, HLA-DPB1, HLA-DQA1, HLA-DQB1, HLA-DQB2, HLA-DRA, HLA-DRB1, HLA-DRB5) gene expression ($\log_2$(TPM+1)) across tumor subtypes in the CCMA cohort. B) Heatmap showing the median expression and unsupervised hierarchical clustering of HLA class I and II genes for different cancer types in CCMA. The color scale represents per-gene z-score normalization, where red indicates higher relative expression and blue denotes lower expression. Tumor categories are annotated on the left.

Figure S4. Antigen presentation pathway activity comparison between molecularly defined cancer subtypes in H3K27M-DMG (A) and ATRT (B). Related to Figure 5 Student t test was used to determine the statistical difference between the pathway scores of DMG_H3.1K27M and DMG_H3.3K27M (A), ATRT Group1 and Group2 (B). Statistical significance is indicated (*p <0.05).

Figure S5. Comparison of HLA gene expression between tumor tissues and cell line models. Related to Figure 6 Expression of HLA class I (HLA-A, HLA-B, HLA-C) and HLA class II (HLA- DQ, HLA-DR, HLA-DP subunits) genes are shown in ridge plots between CCMA cohort (red), OpenPedCan tumor tissues (green) and derived cancer cell lines (blue).

Figure S6. Comparison of pathway activity across cancer subtypes and sample types. Related to Figure 6 and Table S6. (A) Box plots displaying GSVA pathway activity scores for APP, APP-HSP, HLA class I, proteasome, T cell, and TF pathways across pediatric cancer subtypes in CCMA cell lines (orange), OpenPedCan-derived cell lines (purple), and OpenPedCan solid tumor samples (green). Statistical significance from two-way ANOVA is indicated in each panel, assessing differences in pathway activity across cancer subtypes and sample types. (B) Box plots displaying GSVA pathway activity scores for mesenchymal (MES) and adrenergic (ARDN) gene signatures 52 in CCMA neuroblastoma models.

Supplementary table legend

Table S1. HLA class I typing for CCMA cell line models. Related to Figure 1.
1.1 HLA class I typing determined by OptiType, supertypes, zygosity, and cell line metadata
1.2 Detailed concordance status of 28 models with mismatched WGS and RNA-seq inferred HLA types

Table S2. Accuracy of HLA typing and zygosity. Related to Figure 2 and 3.
2.1 HLA prediction accuracy validated with NGS high resolution typing from 24 models
2.2 Homozygosity/heterozygosity detection accuracy of HLA

Table S3. Predicted neoepitopes for CCMA cell line models. Related to Figure 4.
3.1 Predicted neoepitopes with IC50 <500 nM binding affinity from missense, inframe insertion/deletions, and frameshift mutations determined by the pVACseq pipeline

3.2 Predicted neoepitopes with IC50 <500 nM binding affinity from alternative splicing determined by the pVACsplice pipeline

3.3 Predicted neoepitopes with IC50 <500 nM binding affinity from fusion mutations determined by the pVACfuse pipeline

3.4 Commonly predicted neoepitopes with Pediatric Cancer Genome Project cohort by Chang et al., 2017 [S1]

3.5 Commonly predicted neoepitopes with IEDB

Table S4. Pathway Enrichment Analysis between ATRT subtypes (Group 1 vs Group 2). Related to Figure 5.

Table S5. Two-way ANOVA analysis between cancer types and sample formats. Related to Figure 6.

Table S6. Key gene sets used in the pathway analysis. Related to Figure 5 and S6.

Supplemental references

1. Chang, T.-C., Carter, R.A., Li, Y., Li, Y., Wang, H., Edmonson, M.N., Chen, X., Arnold, P., Geiger, T.L., Wu, G., et al. (2017). The neoepitope landscape in pediatric cancers. Genome Medicine 9. 10.1186/s13073-017-0468-3.

Figure S1

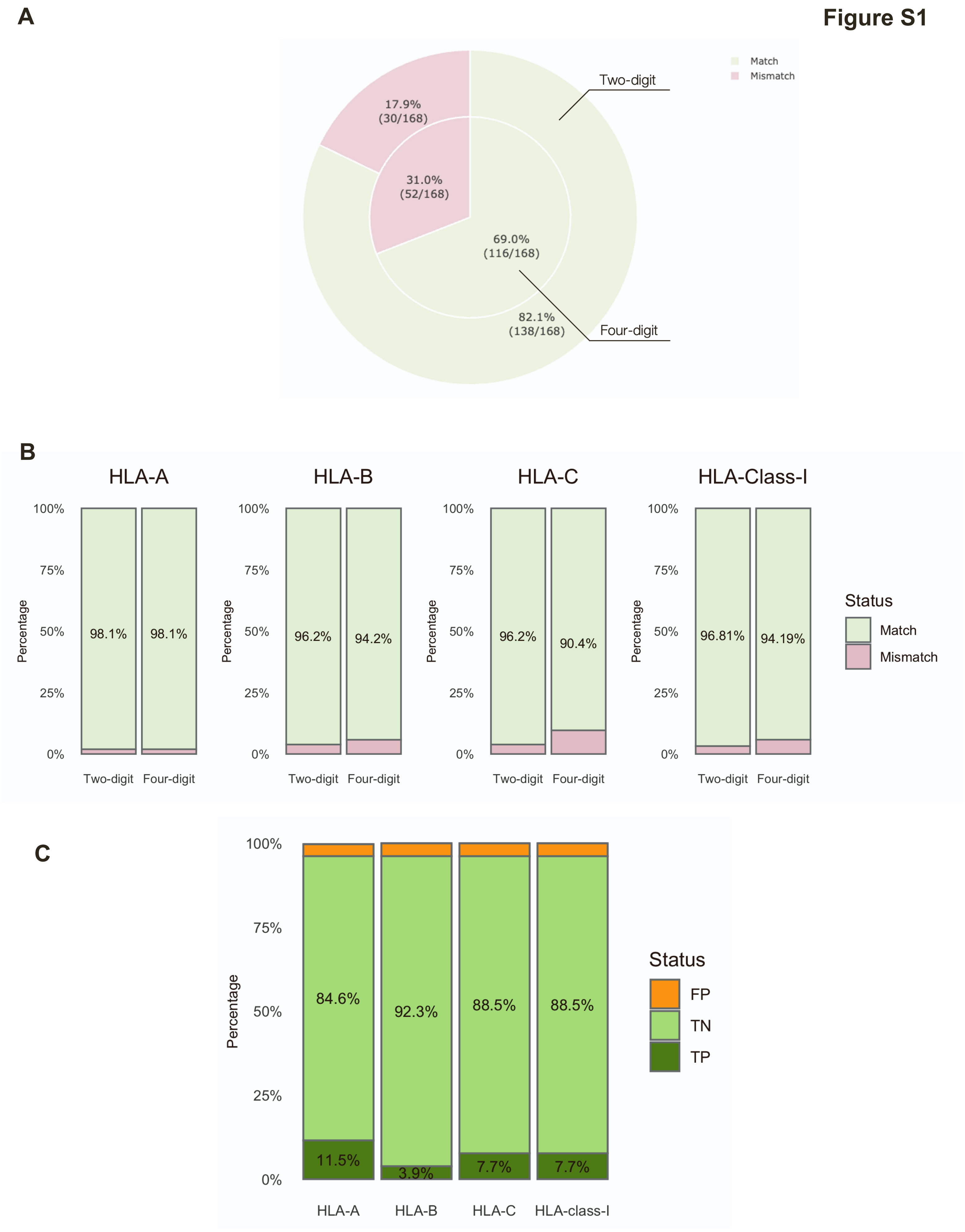
A
Match
Mismatch
Two-digit
Four-digit
17.9%
(30/168)
31.0%
(52/168)
69.0%
(116/168)
82.1%
(138/168)
B
HLA-A
HLA-B
HLA-C
HLA-Class-I
Percentage
100%
75%
50%
25%
0%
98.1%
98.1%
96.2%
94.2%
96.2%
90.4%
96.81%
94.19%
Two-digit
Four-digit
Status
Match
Mismatch
C
Percentage
100%
75%
50%
25%
0%
84.6%
92.3%
88.5%
88.5%
11.5%
3.9%
7.7%
7.7%
HLA-A
HLA-B
HLA-C
HLA-class-I
Status
FP
TN
TP

Figure S2

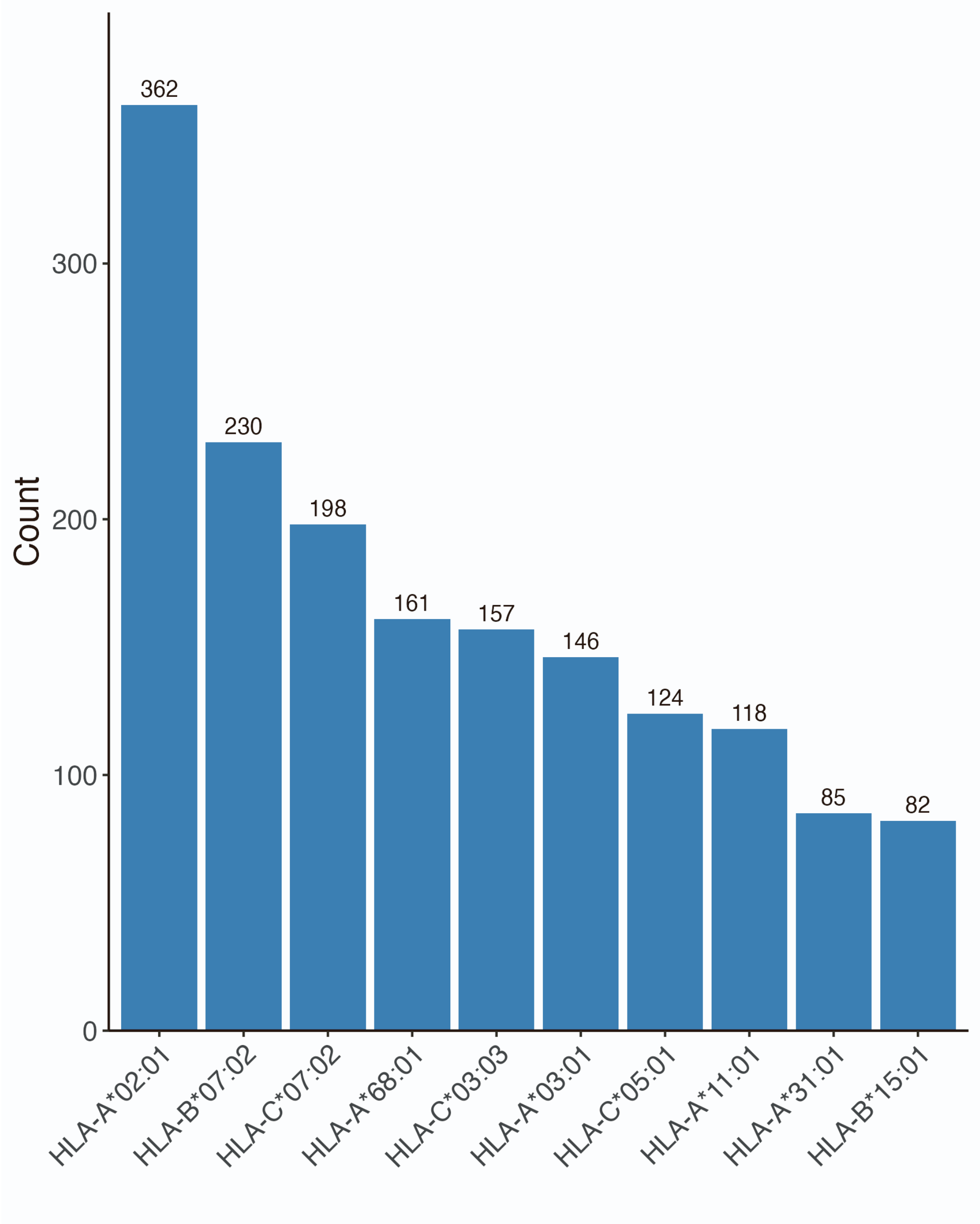

Count
300
200
100
0
362
230
198
161
157
146
124
118
85
82
HLA-A*02:01
HLA-B*07:02
HLA-C*07:02
HLA-A*68:01
HLA-C*03:03
HLA-A*03:01
HLA-C*05:01
HLA-A*11:01
HLA-A*31:01
HLA-B*15:01

Figure S3

A

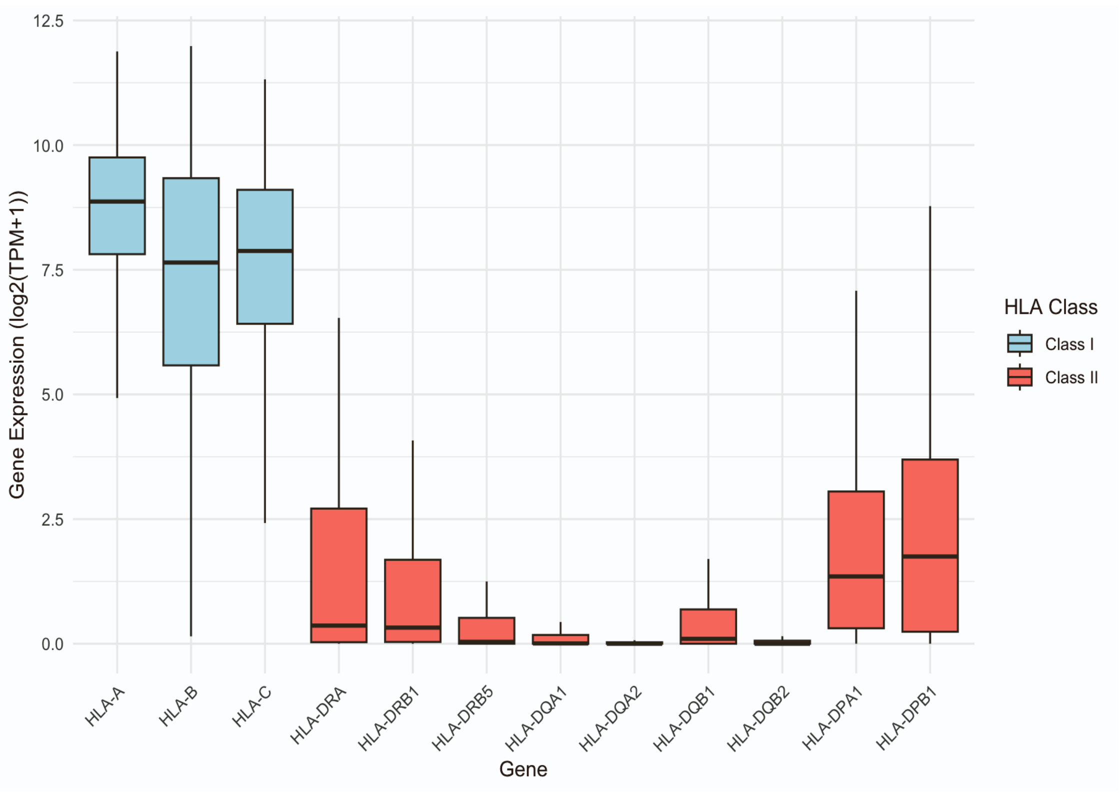

12.5
10.0
7.5
5.0
2.5
0.0
Gene Expression (log2(TPM+1))
HLA-A
HLA-B
HLA-C
HLA-DRA
HLA-DRB1
HLA-DRB5
HLA-DQA1
HLA-DQA2
HLA-DQB1
HLA-DQB2
HLA-DPA1
HLA-DPB1
Gene
HLA Class
Class I
Class II

B

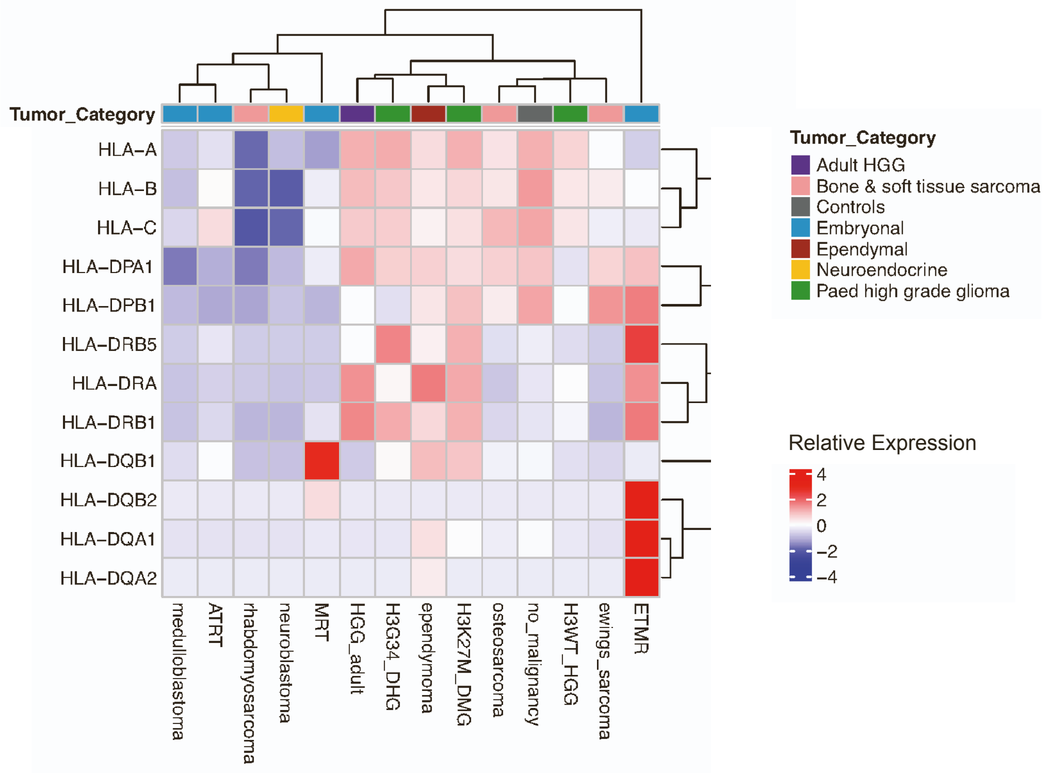

Tumor_Category
HLA−A
HLA−B
HLA−C
HLA−DPA1
HLA−DPB1
HLA−DRB5
HLA−DRA
HLA−DRB1
HLA−DQB1
HLA−DQB2
HLA−DQA1
HLA−DQA2
medulloblastoma
ATRT
rhabdomyosarcoma
neuroblastoma
MRT
HGG_adult
H3G34_DHG
ependymoma
H3K27M_DMG
osteosarcoma
no_malignancy
H3WT_HGG
ewings_sarcoma
ETMR
Tumor_Category
Adult HGG
Bone & soft tissue sarcoma
Controls
Embryonal
Ependymal
Neuroendocrine
Paed high grade glioma
Relative Expression
4
2
0
−2
−4

Figure S4

A

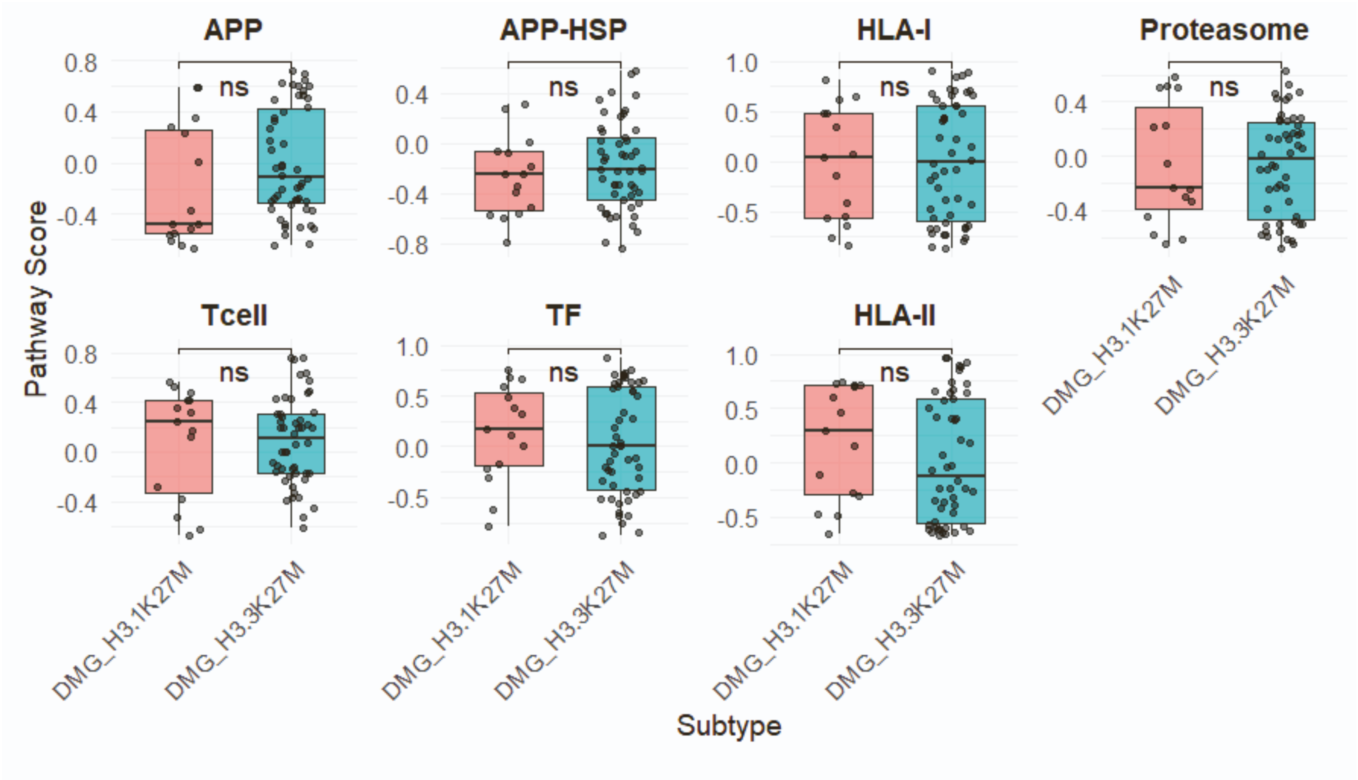

APP
APP-HSP
HLA-I
Proteasome
Tcell
TF
HLA-II
ns
Pathway Score
DMG_H3.1K27M
DMG_H3.3K27M
Subtype

B

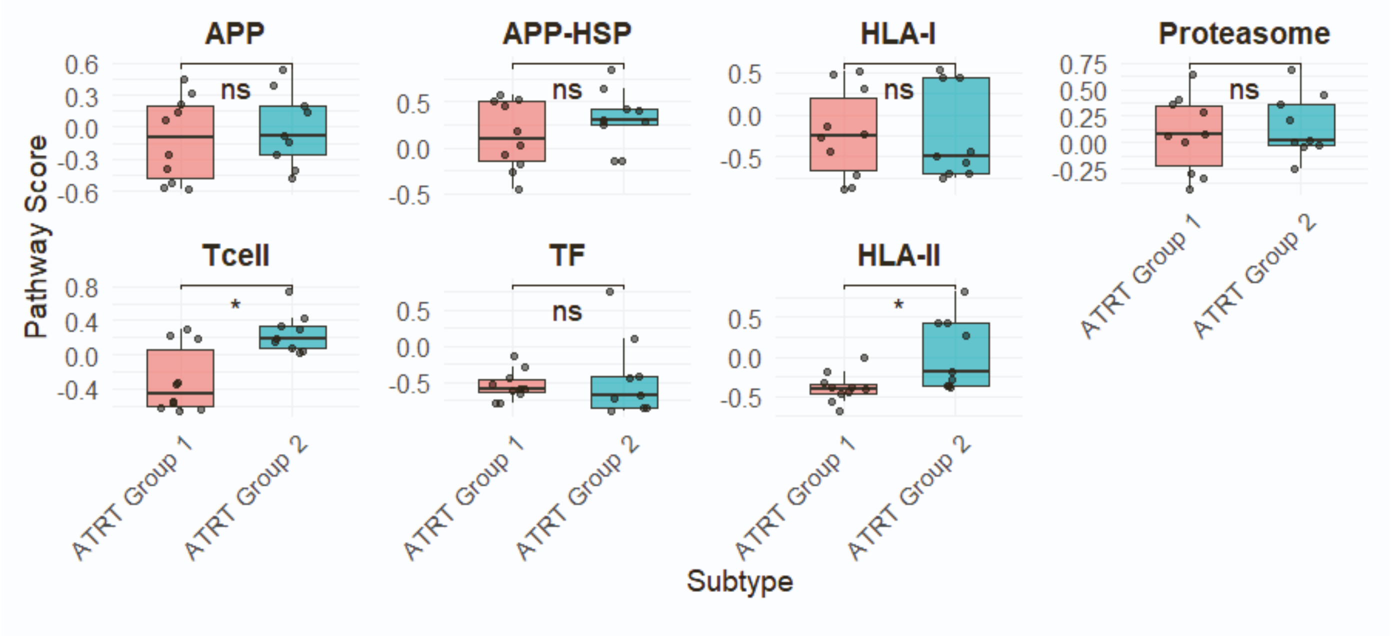

APP
APP-HSP
HLA-I
Proteasome
Tcell
TF
HLA-II
ns
*
Pathway Score
ATRT Group 1
ATRT Group 2
Subtype

Figure S5

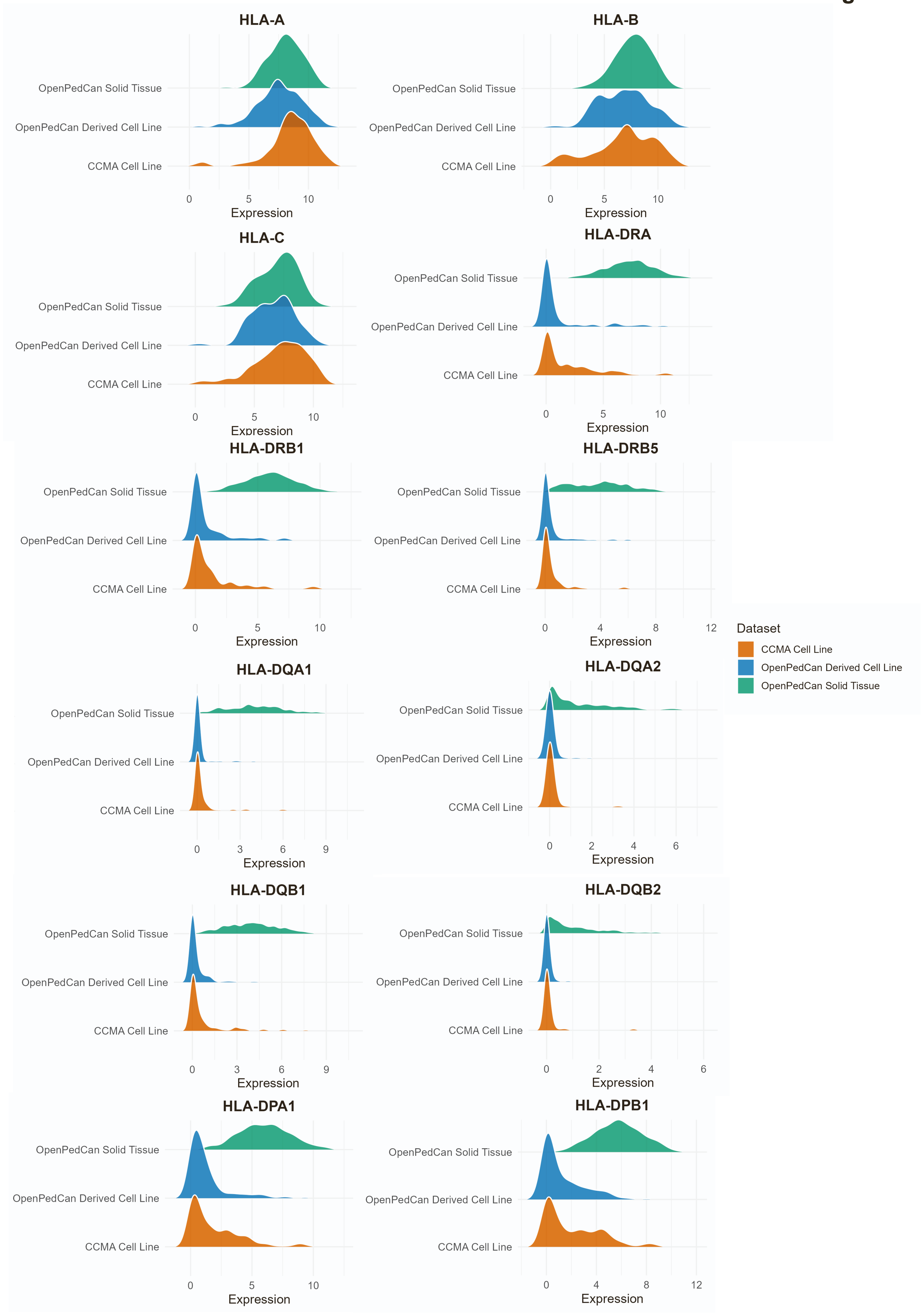
HLA-A
HLA-B
HLA-C
HLA-DRA
HLA-DRB1
HLA-DRB5
HLA-DQA1
HLA-DQA2
HLA-DQB1
HLA-DQB2
HLA-DPA1
HLA-DPB1
OpenPedCan Solid Tissue
OpenPedCan Derived Cell Line
CCMA Cell Line
Expression
Dataset
CCMA Cell Line
OpenPedCan Derived Cell Line
OpenPedCan Solid Tissue

Figure S6

A

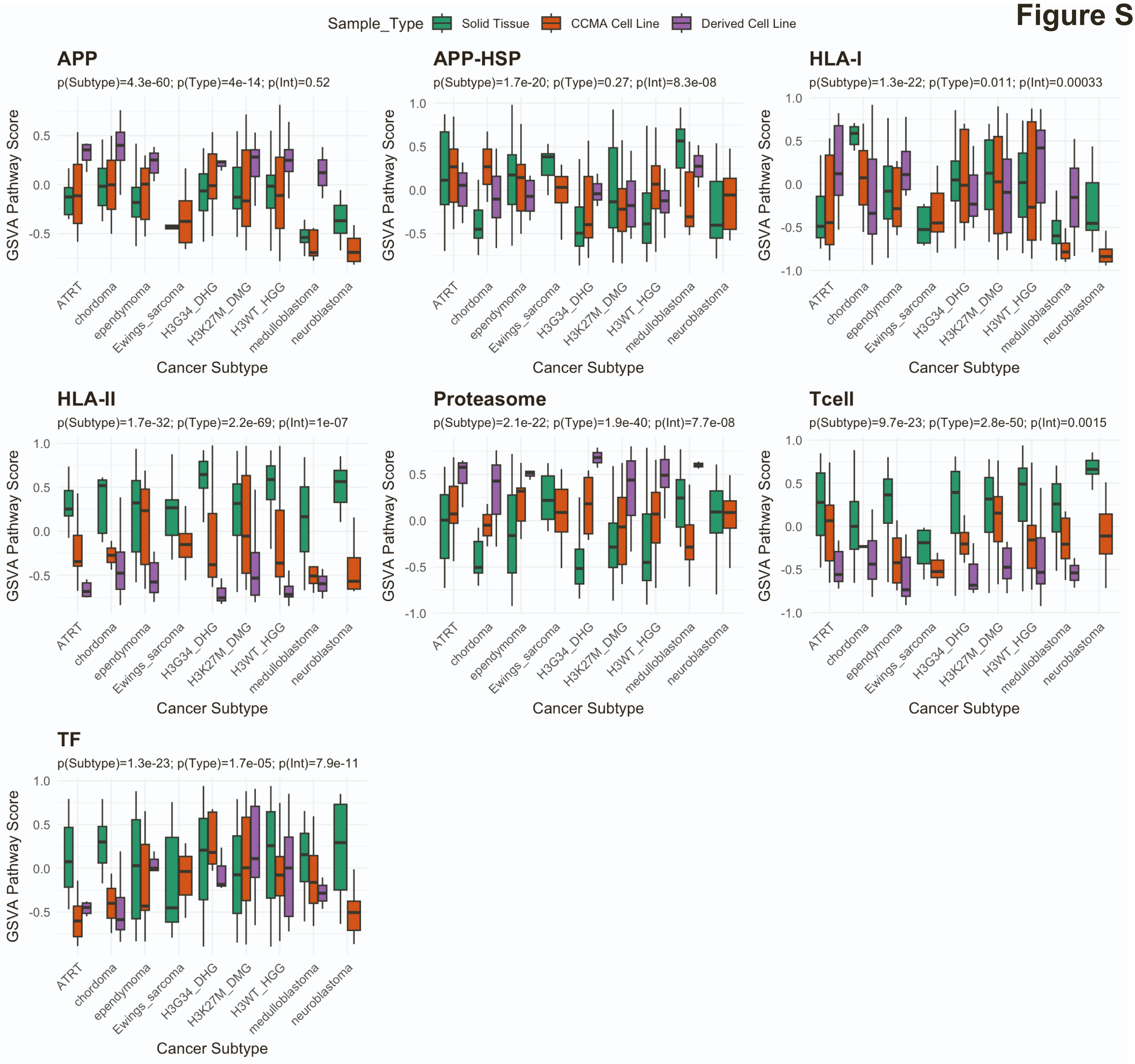
Sample_Type
Solid Tissue
CCMA Cell Line
Derived Cell Line
APP
p(Subtype)=4.3e-60; p(Type)=4e-14; p(Int)=0.52
APP-HSP
p(Subtype)=1.7e-20; p(Type)=0.27; p(Int)=8.3e-08
HLA-I
p(Subtype)=1.3e-22; p(Type)=0.011; p(Int)=0.00033
HLA-II
p(Subtype)=1.7e-32; p(Type)=2.2e-69; p(Int)=1e-07
Proteasome
p(Subtype)=2.1e-22; p(Type)=1.9e-40; p(Int)=7.7e-08
Tcell
p(Subtype)=9.7e-23; p(Type)=2.8e-50; p(Int)=0.0015
TF
p(Subtype)=1.3e-23; p(Type)=1.7e-05; p(Int)=7.9e-11
GSVA Pathway Score
Cancer Subtype
ATRT
chordoma
ependymoma
Ewings_sarcoma
H3G34_DHG
H3K27M_DMG
H3WT_HGG
medulloblastoma
neuroblastoma
1.0
0.5
0.0
-0.5
-1.0

B

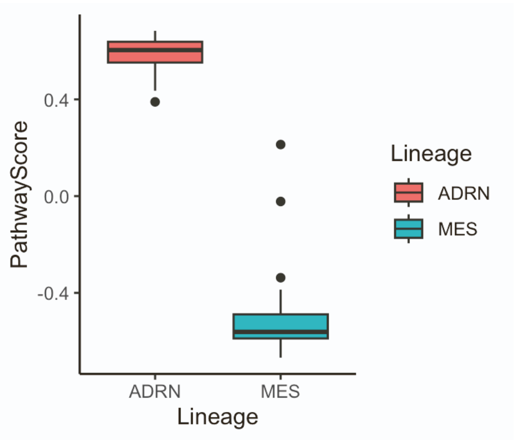
PathwayScore
0.4
0.0
-0.4
ADRN
MES
Lineage
Lineage
ADRN
MES

Table S4

Pathway	p_value	FDR
app	0.5843	0.6678
apphsp	0.959	0.959
hla1	0.5353	0.6678
hla3	0.2793	0.5586
proteasome	0.0629	0.2516
tcell	0.0036	0.0288
tf	0.2582	0.5586
hla2	0.5353	0.6678

Table S5

Gene	CCMA_Class_p_value	Sample_Type_p_value	Interaction_p_value
HLA-A	2.29E-40	0.013601798	0.017971726
HLA-B	4.62E-42	4.61E-11	2.65E-11
HLA-C	2.40E-46	0.539805556	4.92E-06
HLA-DPA1	2.61E-45	2.14E-104	0.234594079
HLA-DPB1	1.80E-33	2.77E-94	0.04434816
HLA-DQA1	2.03E-52	5.26E-123	0.012464829
HLA-DQA2	2.56E-24	7.91E-41	0.001901612
HLA-DQB1	1.20E-35	1.84E-107	0.013018857
HLA-DQB2	4.60E-14	1.10E-41	0.005873032
HLA-DRA	1.22E-57	5.93E-130	0.105834421
HLA-DRB1	2.59E-56	1.83E-125	0.020817124
HLA-DRB5	2.78E-49	2.11E-103	0.000133718

Table S6

Name	Group
CANX	APP
CALR	APP
ERAP1	APP
ERAP2	APP
IFI30	APP
IFNG	APP
KLRC1	APP
KLRC2	APP
KLRC3	APP
PDIA3	APP
TAPBP	APP
TAPBPL	APP
TAP1	APP
TAP2	APP
HSP90AA1	APP-HSP
HSP90AB1	APP-HSP
HSPA1L	APP-HSP
HSPA1A	APP-HSP
HSPA1B	APP-HSP
HSPA2	APP-HSP
HSPA4	APP-HSP
HSPA5	APP-HSP
HSPA6	APP-HSP
HSPA8	APP-HSP
B2M	HLA1
HLA-A	HLA1
HLA-B	HLA1
HLA-C	HLA1
HLA-E	HLA1
HLA-F	HLA1
HLA-G	HLA1
HLA-H	HLA1
HLA-DMA	HLA2
HLA-DMB	HLA2
HLA-DOA	HLA2
HLA-DOB	HLA2
HLA-DPA1	HLA2
HLA-DPB1	HLA2
HLA-DQA1	HLA2
HLA-DQA2	HLA2
HLA-DQB1	HLA2
HLA-DQB2	HLA2

HLA-DRA	HLA2
HLA-DRB1	HLA2
HLA-DRB3	HLA2
HLA-DRB4	HLA2
HLA-DRB5	HLA2
TNF	HLA3
ECPAS	proteasome
PSMB10	proteasome
PSMB4	proteasome
PSMB5	proteasome
PSMB6	proteasome
PSMB7	proteasome
PSMB8	proteasome
PSMB9	proteasome
PSME1	proteasome
PSME2	proteasome
PSME3	proteasome
PSME4	proteasome
PSMG2	proteasome
PSMG3	proteasome
PSMG4	proteasome
POMP	proteasome
PSMB11	proteasome
CD4	Tcell
CD74	Tcell
CD8A	Tcell
CD8B	Tcell
CREB1	TF
CIITA	TF
ICAM1	TF
